# Facile synthesis of Pd@graphene nanocomposites with enhanced catalytic activity towards Suzuki coupling reaction

**DOI:** 10.1038/s41598-020-68124-w

**Published:** 2020-07-16

**Authors:** Mujeeb Khan, Mohammed Rafi Shaik, Syed Farooq Adil, Mufsir Kuniyil, Muhammad Ashraf, Hajo Frerichs, Massih Ahmad Sarif, Mohammed Rafiq H. Siddiqui, Abdulrahman Al–Warthan, Joselito P. Labis, Mohammad Shahidul Islam, Wolfgang Tremel, Muhammad Nawaz Tahir

**Affiliations:** 1 Department of Chemistry, College of Science, King Saud University, P.O. Box 2455, Riyadh, 11451 Kingdom of Saudi Arabia; 2 Department of Chemistry, Koneru Lakshmaiah Education Foundation, Vaddeswaram, Guntur, Andhra Pradesh 522502 India; 3 Department of Chemistry, King Fahd University of Petroleum and Minerals, P.O. Box 5048, Dhahran, 31261 Kingdom of Saudi Arabia; 4 Institut für Anorganische Chemie Und Analytische Chemie, Johannes Gutenberg-Universität, Duesbergweg 10-14, 55128 Mainz, Germany; 5 King Abdullah Institute for Nanotechnology, King Saud University, Riyadh, 11451 Kingdom of Saudi Arabia

**Keywords:** Chemistry, Materials science, Nanoscience and technology

## Abstract

A facile and chemical specific method to synthesize highly reduced graphene oxide (HRG) and Pd (HRG@Pd) nanocomposite is presented. The HRG surfaces are tailored with amine groups using 1-aminopyrene (1-AP) as functionalizing molecules. The aromatic rings of 1-AP sit on the basal planes of HRG through π–π interactions, leaving amino groups outwards (similar like self-assembled monolayer on 2D substrates). The amino groups provide the chemically specific binding sites to the Pd nucleation which subsequently grow into nanoparticles. HRG@Pd nanocomposite demonstrated both uniform distribution of Pd nanoparticles on HRG surface as well as excellent physical stability and dispersibility. The surface functionalization was confirmed using, ultraviolet–visible (UV–Vis), Fourier transform infra-red and Raman spectroscopy. The size and distribution of Pd nanoparticles on the HRG and crystallinity were confirmed using high-resolution transmission electron microscopy and powder X-ray diffraction and X-ray photoelectron spectroscopy. The catalytic efficiency of highly reduced graphene oxide-pyrene-palladium nanocomposite (HRG-Py-Pd) is tested towards the Suzuki coupling reactions of various aryl halides. The kinetics of the catalytic reaction (Suzuki coupling) using HRG-Py-Pd nanocomposite was monitored using gas chromatography (GC).

## Introduction

The highly reduced graphene oxide (HRG) with its exceptional physicochemical properties is among extensively studied materials in the world^1,2^. It is the strongest, thinnest and stiffest material with several remarkable properties, including high thermal and electric conductivities and large theoretical specific surface area^3,4^. These unique properties have attracted the vigil eye of researchers in both scientific (academics) and engineering communities (industrial applications) ^5^. Currently, several methods have been applied to obtain bulk quantities of defect free graphene, which are mainly classified into the *bottom-up* and *top-down* approaches^6,7^. The most popular methods under the *bottom-up* approaches include chemical vapor deposition (CVD), chemical conversion, and arc discharge^8,9^. Whereas, the *top-down* approach involve, the sequential oxidation and reduction of graphite. These chemical methods (*top-down* approaches), offer excellent opportunities for the production of large quantities of graphene like materials, which is best known as highly reduced graphene oxide (HRG)^10,11^.

The recent advancement in the synthesis of homogeneously dispersed graphene using different reduction and functionalization techniques, have led to the development of various graphene based hybrid materials, such as graphene-inorganic nanoparticles (NPs) based nanocomposites^12,13^. The hybridization of inorganic NPs with graphene further enhance the properties and broaden the applications ranging from the medical to the energy sector, including catalysis^14,15^. The catalytic activities of HRGs can be further enhanced either by doping with various heteroatoms or blending them with other nanomaterials to form functional nanocomposites^16–18^. Therefore, the HRGs not only possess the potential to be promising catalysts but also are attractive support materials for developing various hybrid catalysts^19^.

Among inorganic nanomaterials, metallic nanoparticles based nanocomposites takes the central position with range of catalytic applications. Particularly, graphene-palladium (HRG/Pd) nanocomposites have been extensively applied as chemical catalysts for several organic transformations^20,21^. The cooperative effects and intrinsic properties of both HRG and Pd, such as, large surface area of HRG, ample presence of active sites and inherent catalytic properties of Pd collectively contribute to the improvement of the catalytic properties of hybrid nanocatalyst^22^. The HRG/Pd based nanocatalysts were largely explored for various coupling reactions, such as, Heck coupling and Suzuki coupling. For instance, Scheuermann et al. have demonstrated the preparation and application of chemically derived functionalized graphene-palladium nanocomposites for the Suzuki–Miyaura coupling. The composite has exhibited superior catalytic activities with excellent conversions, high turnover frequencies and low palladium leaching when compare to the conventional Pd/C catalyst^23^.

The preparation of metallic NPs and graphene NPs based nanocomposites are commonly achieved either via post or in situ immobilization of metallic nanoparticles onto HRG^24^. The former involves the mixing of separate solutions of graphene and pre-synthesized NPs, whereas, the later required the simultaneous reduction of graphite oxide (GO) or graphene oxide (GRO) and the respective metal salts^25,26^. Graphene-inorganic NPs based nanocomposites obtained from either method; usually suffer from aggregation due to the strong Van der Waals interactions, such as, π–π interactions, low density and non-uniform coverage of inorganic nanoparticles, which adversely effects their potential applications in different fields, including catalysis^27–29^. The development of benign, scalable and reproducible protocols for the synthesis of HRG-metallic nanoparticles is inevitable. Surface functionalization of the NPs and/or graphene is usually carried out^30^ to overcome the aforementioned challenges for the preparation of graphene based nanocomposites.

Among surface functionalization methodologies, a number of stabilizing agents (surfactants, polymers, polycyclic aromatics “π–π stacking” molecules) have been applied for the purpose of non-covalent functionalization to prevent restacking of graphene nanosheets^31,32^. Among various stabilizing agents, polycyclic aromatic hydrocarbons (PAHs), such as, anthracene, tetracene, pyrene, coronene, exhibited excellent potential as stabilizers, due to their strong π–π interactions with the conjugated basal planes of grapheme^33,34^. Among these polycyclic aromatics, pyrene and various pyrene derivatives containing –NH_2_, –COOH, –OH, –SH functional groups have gained prominent attention as stabilizers^35–37^.

Based on our previous experience to synthesize layered transition metal chalcogenides and inorganic nanomaterials based nanocomposites employing Hard Soft Acid Base (HSAB) concept, we demonstrate a novel methodology to synthesize highly reduced graphene oxide-pyrene-palladium nanocomposite (HRG-Py-Pd) nanocomposites using 1-aminopyrene (1-AP) to tailor the HRG surfaces. 1-aminopyrene (1-AP) as stabilizer for this purpose, plays a dual role; (i) the basal plane of 1-AP tailor the surface of HRG through strong π–π interactions, and (ii) the amino groups, to provide a homogeneous matrices for the nucleation and growth of Pd nanoparticles. The as-prepared nanocatalysts were tested for their catalytic activities towards Suzuki–Miyaura coupling reactions in aqueous solution. The catalytic activities of HRG-Py-Pd nanocomposites were compared with graphene-palladium (HRG-Pd) nanocomposites without pyrene. All the nanocomposites prepared and the organic products obtained from the catalytic reactions were characterized using various analytical and microscopic techniques, such as, XRD, UV–Vis, FT-IR, Raman, XPS, and HRTEM.

## Results and discussion

**Scheme 1:**
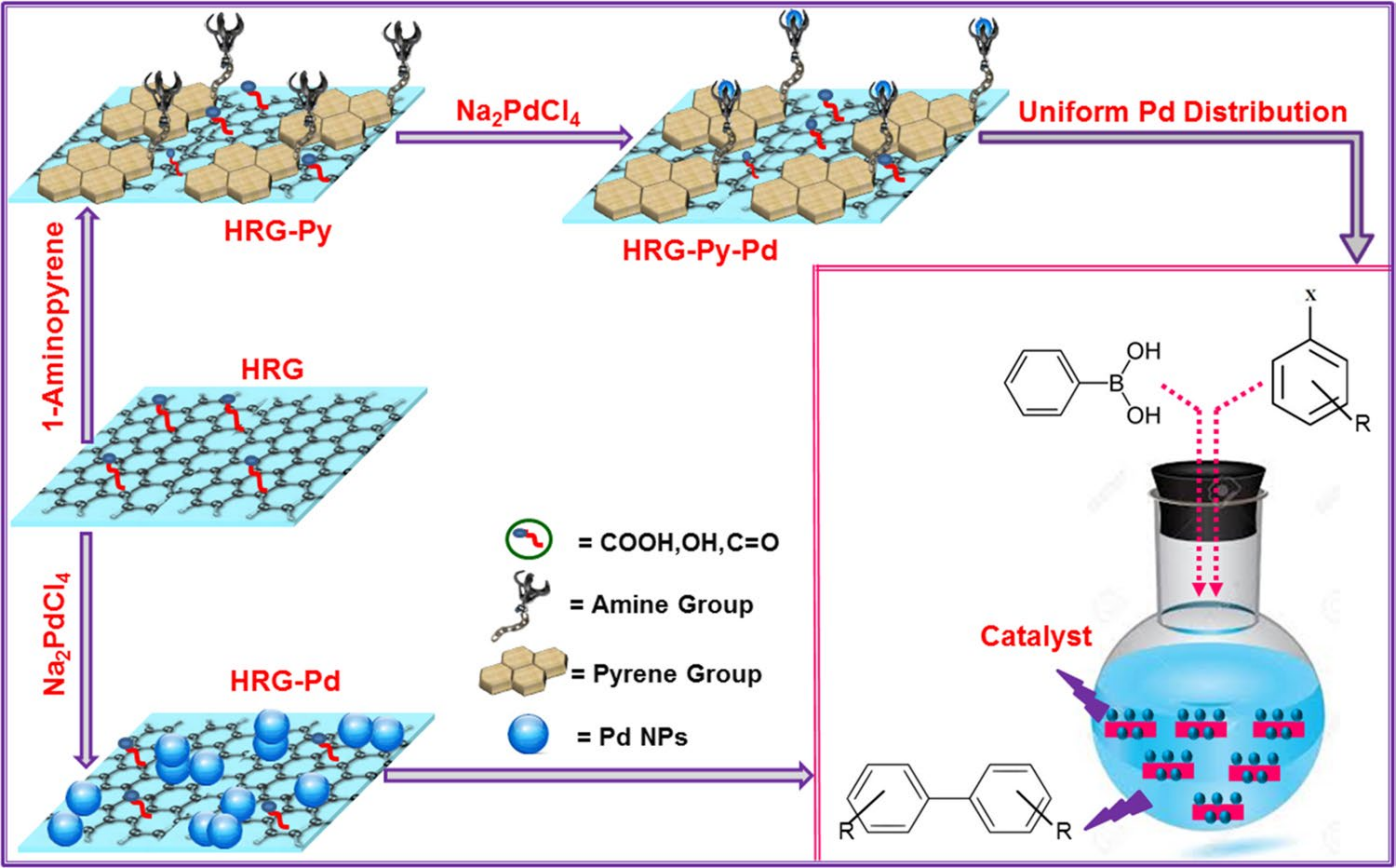
Schematic representation of the preparation of HRG-Py-Pd nanocomposites.

Generally, the catalytic properties of graphene based nanocomposites are adversely affected by the irreversible agglomeration of graphene nanosheets due to their strong van der Waals interactions. To minimize the influence, we demonstrate an approach to noncovalently functionalize the surface of graphene with polycyclic aromatic hydrocarbons (PAHs) with dual function. The overall methodology to prepare HRG-Py-Pd nanocomposites and its catalytic application is depicted in Scheme 1. Briefly, the graphene oxide (GO) was prepared using a modified Hummer method, which was then reduced by hydrazine hydrate to obtain highly reduced graphene oxide (HRG). Subsequently, the HRG and 1-AP were sonicated together in methanol to obtain graphene-pyrene composite (HRG-Py). The 1-AP have excellent ability to strongly anchor the planar surface of graphene nanosheets whereas the head group (-NH_2_) helps binding the nucleates of Pd, leading to homogeneous growth of nanoparticles. The as-prepared HRG-Py-Pd nanocomposite was used as a catalyst for the Suzuki coupling reactions. Furthermore, its catalytic activity was also compared with graphene-palladium (HRG-Pd) nanocomposite prepared without 1-AP.

### UV–Vis and FT-IR analysis

**Figure 1:**
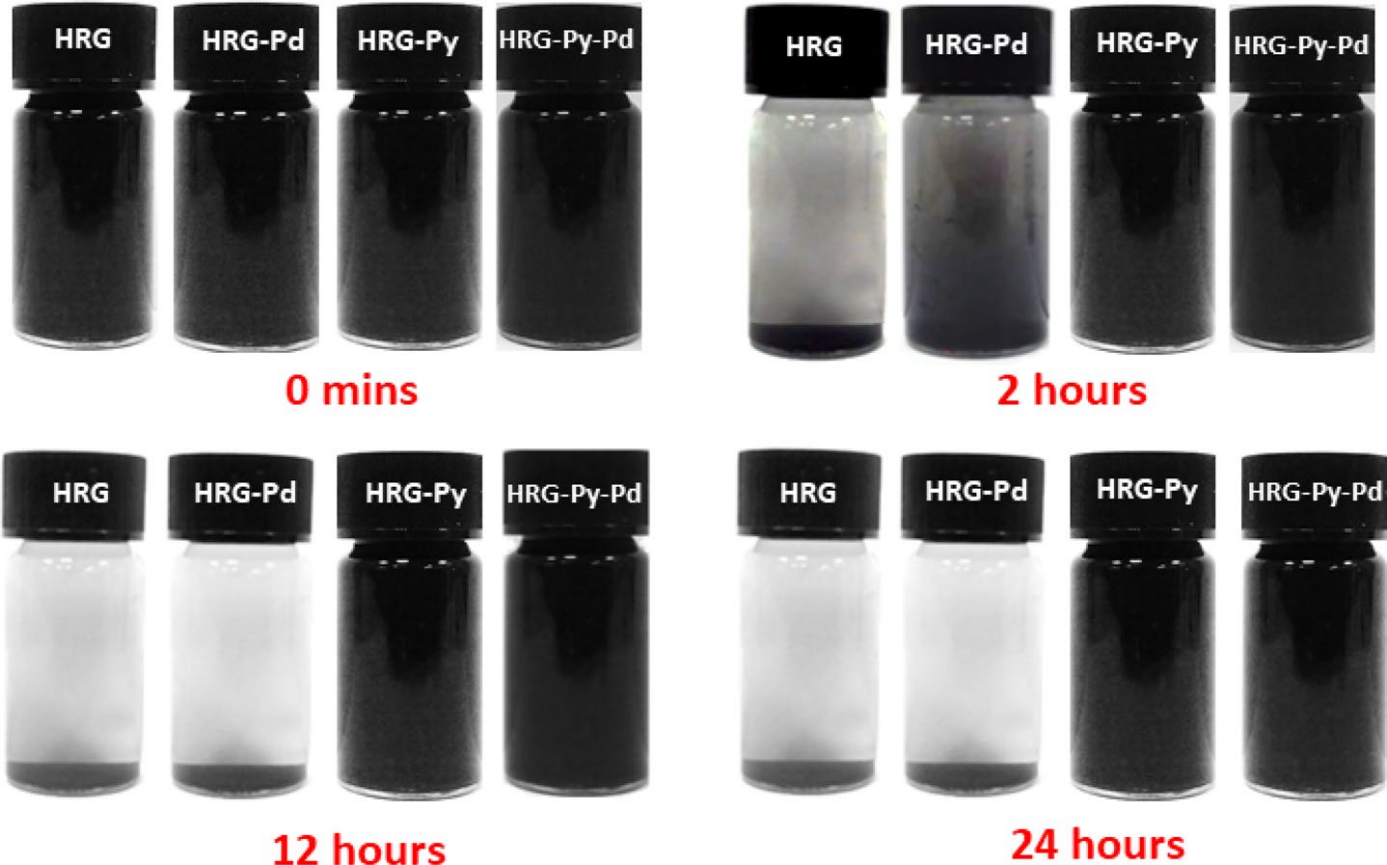
Digital images of the dispersions of HRG, HRG-Pd, HRG-Py and HRG-Py-Pd.

The stabilizing quality of 1-AP was tested by investigating and comparing the dispersibilities of HRG-Py, HRG-Py-Pd with that of HRG and HRG-Pd in aqueous solution. For this purpose, the dispersions were prepared by sonicating 5 mg of each sample in 10 ml of water. The result indicates that both HRG-Py and HRG-Py-Pd have demonstrated superior dispersions in aqueous solution when compared with HRG and HRG-Pd as shown in Fig. 1. This clearly indicates that 1-AP has greatly enhanced the dispersibility of both graphene and graphene-Pd nanocomposite.

**Figure 2:**
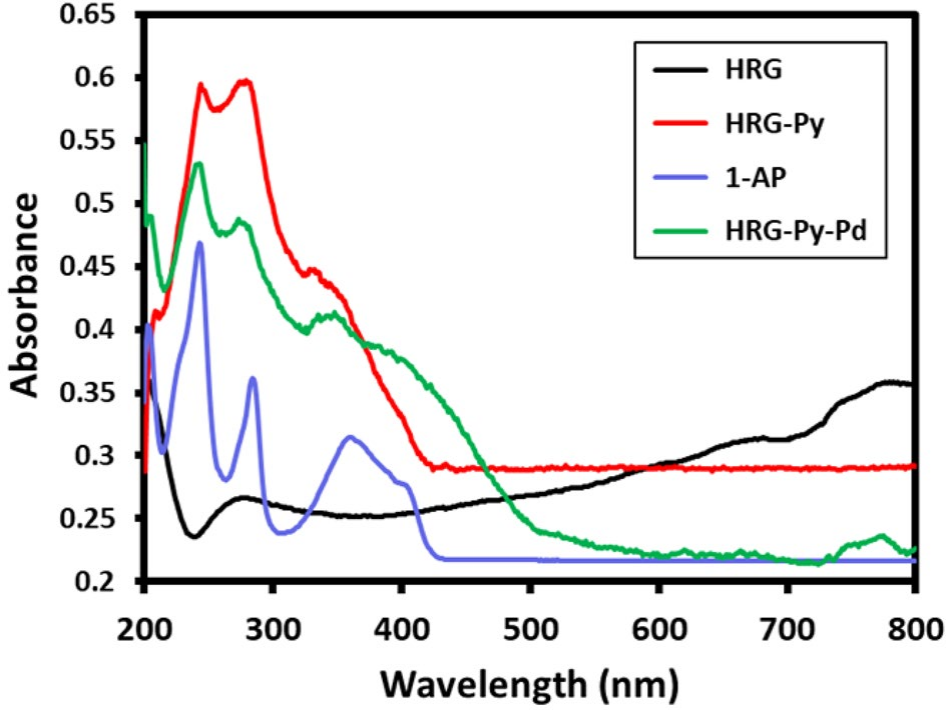
UV–Vis absorption spectra of 1-Aminopyrene (1-AP, blue line), highly reduced graphene oxide (HRG, black line), graphene-pyrene composite (HRG-Py, red line), and graphene-pyrene-Pd (HRG-Py-Pd, green line).

**Figure 3:**
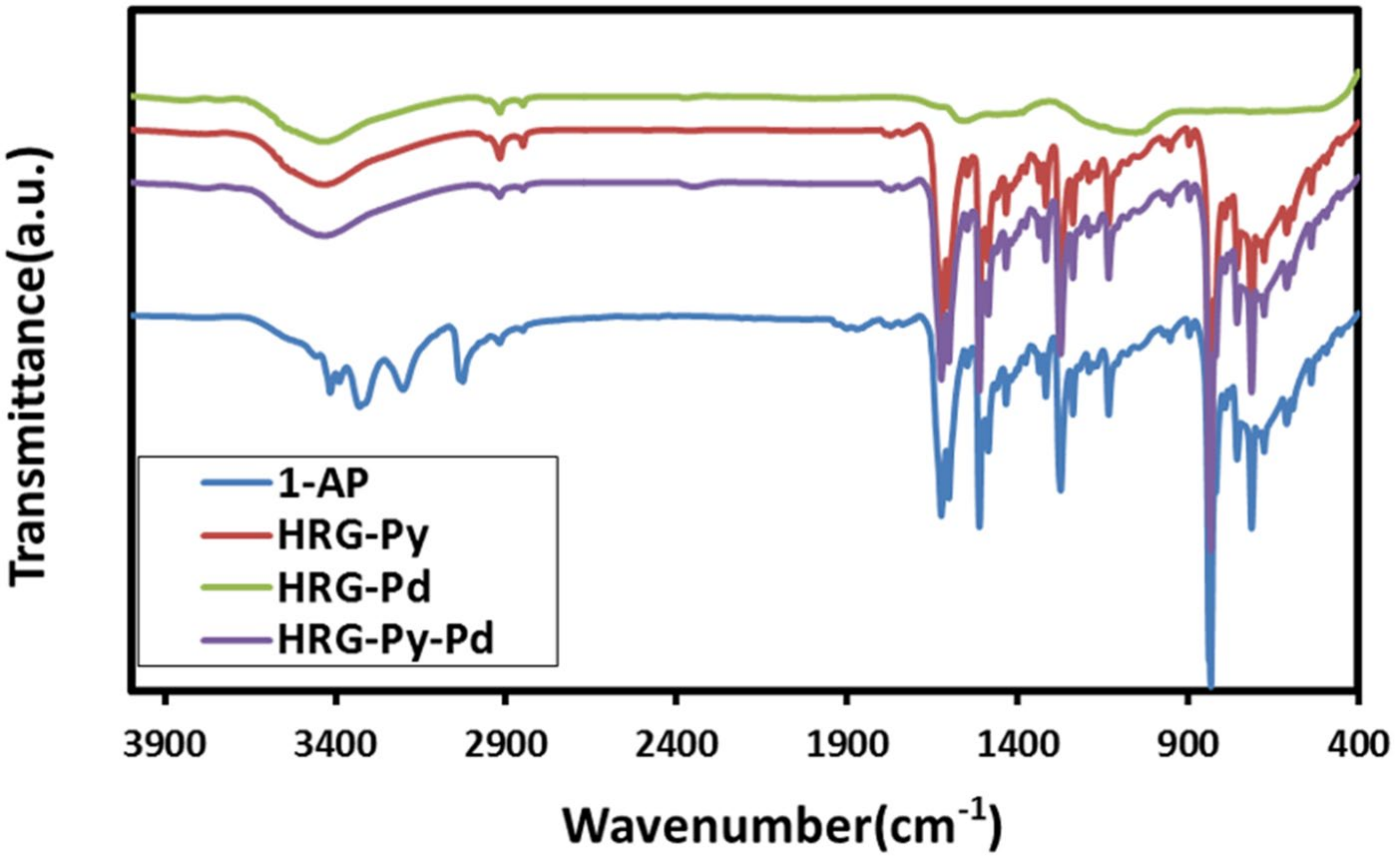
FT-IR spectra of 1-Aminopyrene (1-AP, blue line), graphene-pyrene composite (HRG-Py, red line), highly reduced graphene-palladium nanocomposite (HRG-Pd, green line) and graphene-pyrene-Pd nanocomposite (HRG-Py-Pd, purple line).

The adsorption of 1-AP on the surface of HRG was initially confirmed using UV–Vis spectroscopy by comparing the UV–Vis spectra of 1-AP, HRG, HRG-Py and HRG-Py-Pd as shown in Fig. 2. The characteristic absorption bands of 1-AP appear at ~ 242, ~ 285 and 360 nm (blue line Fig. 2, whereas, the HRG has a typical absorption band at ~ 270 nm (black line, Fig. 2). Notably, the existence of bands at ~ 245, ~ 282 and ~ 355 nm in the spectra of both HRG-Py and HRG-Py-Pd (red and green lines, Fig. 2) clearly suggest the presence of 1-AP on the surface of HRG. Furthermore, this is also confirmed by the absence of these peaks in the UV spectrum of HRG-Pd (Data not shown), which exhibited a typical featureless spectrum. Evidently, the absorption bands in both HRG-Py and HRG-Py-Pd become broader due to the strong π-π interactions between the pyrenyl group of 1-AP and the basal plane of HRG. This noncovalent functionalization of HRG by 1-AP was further confirmed by FT-IR analysis as shown in Fig. 3. Although, some of the peaks are unresolved in the FT-IR spectrum of both HRG-Py and HRG-Py-Pd, the similarities between these two spectra and their differences with that of the IR spectrum of HRG-Pd, strongly suggests the presence of 1-AP on the surface of HRG nanosheets in these samples. For instance, the absorption peaks in the range of 800 to 1,700 cm^−1^ belonging to the aromatics of 1-AP is also present in both HRG-Py and HRG-Py-Pd, however, no peaks were observed in the similar range in the FT-IR spectrum of HRG-Pd, which clearly suggested the absence of 1-AP in this sample.

**Figure 4:**
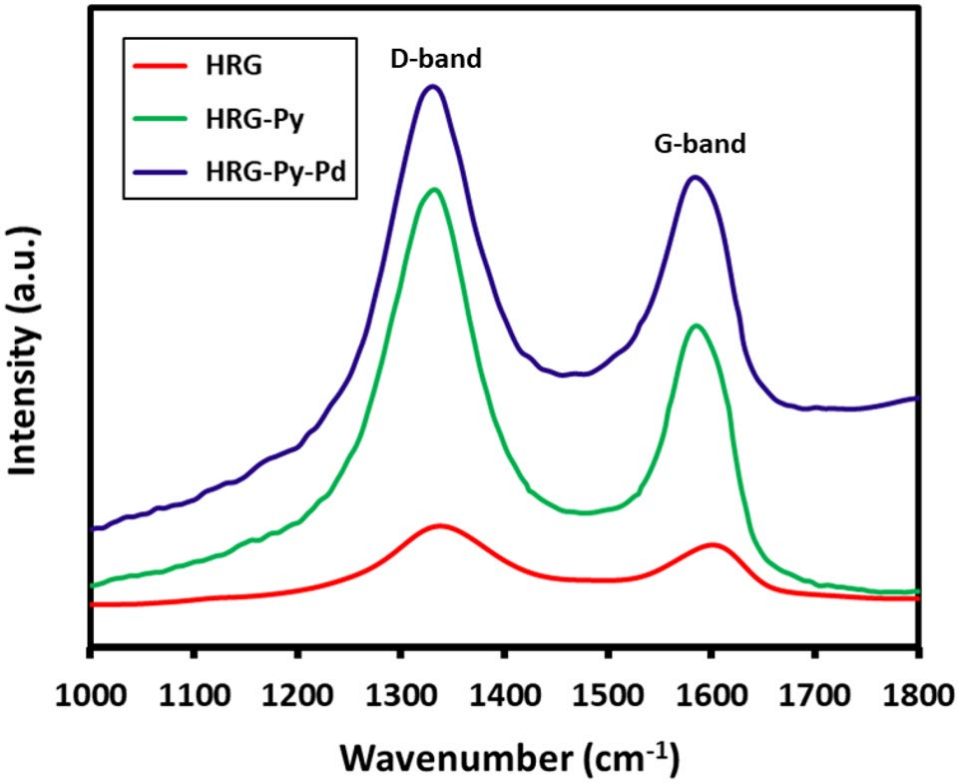
Raman analysis of HRG (red line), HRG-Py (green line) and HRG-Py-Pd (blue line).

Raman spectroscopy was applied to monitor the reduction of GRO. Raman spectra of HRG, HRG-Py and HRG-Py-Pd are displayed in Fig. 4. The HRG spectrum (red line), shows the G and D bands centered at 1589 cm^−1^ and 1,345 cm^−1^, respectively. The G band after functionalization with 1-AP becomes narrower which confirm the more ordered and *SP*^2^ character of carbon support. However, upon Formation of HRG-Py-Pd nanocomposites, the G and D bands centered at 1585 and 1,332 cm^−1^, respectively. The G band again become little broader as compared to HRG-Py which shows little defects. The emergence of some visible changes in the Raman spectra of HRG after growth of Pd reflects the formation of HRG-Py-Pd nanocomposites.

### XRD analysis

**Figure 5:**
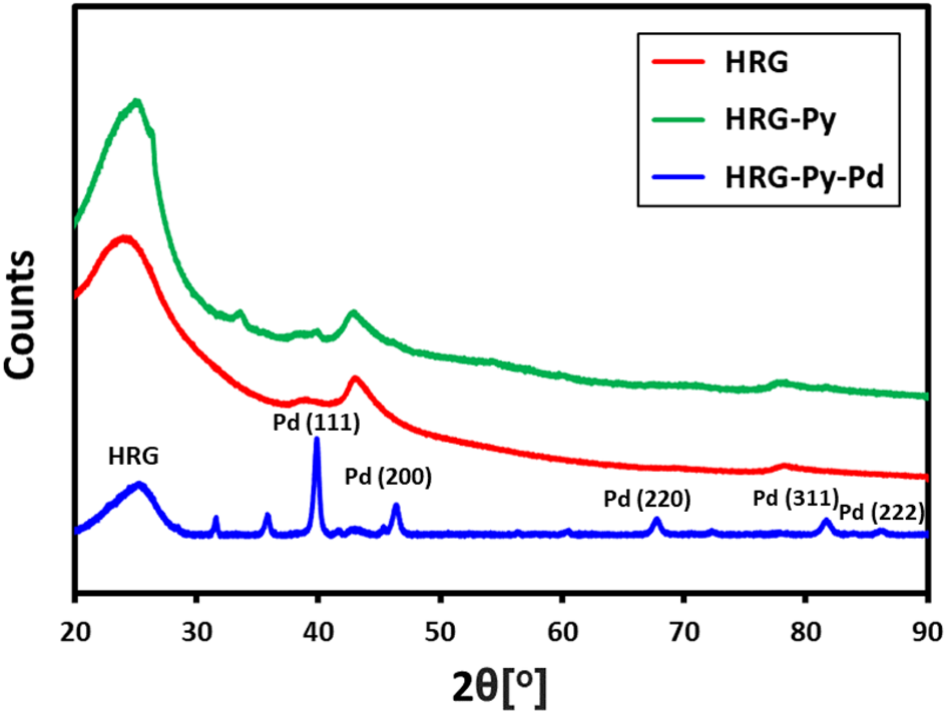
XRD diffractograms of highly reduced graphene (HRG, red line), graphene-pyrene composite (HRG-Py, green line), and graphene-pyrene-Pd nanocomposite (HRG-Py-Pd, blue line).

The crystallinity and phase purity of the HRG-Py-Pd and HRG-Pd was confirmed by XRD analysis. The XRD diffractogram of HRG, HRG-Py, HRG-Py-Pd are given in Fig. 5 and the XRD diffractogram of HRG-Pd given in supplementary file (Fig. S1). A broad reflection at 2θ = 22.4°, which is the characteristic reflection of HRG is present in all these diffractogram. In addition to this reflection, both HRG-Py-Pd and HRG-Pd also exhibit several other reflections at 40.02° (111), 46.49° (200), 68.05° (220), 81.74° (311) and 86.24° (222), which correspond to the Pd NPs^38^. These reflections, apart from the characteristic reflections of HRG in the XRD diffractograms of both HRG-Py-Pd and HRG-Pd can be indexed to face centered cubic (*fcc*) structure of Pd (JCPDS: 87–0,641, space group: Fm3m (225)). On the basis of the half width of the most intense peak at 40.02° (111) reflection, the average crystallite size (∼5 nm) of the Pd NPs was determined using the Scherrer equation ^39^.

### XPS analysis

**Figure 6:**
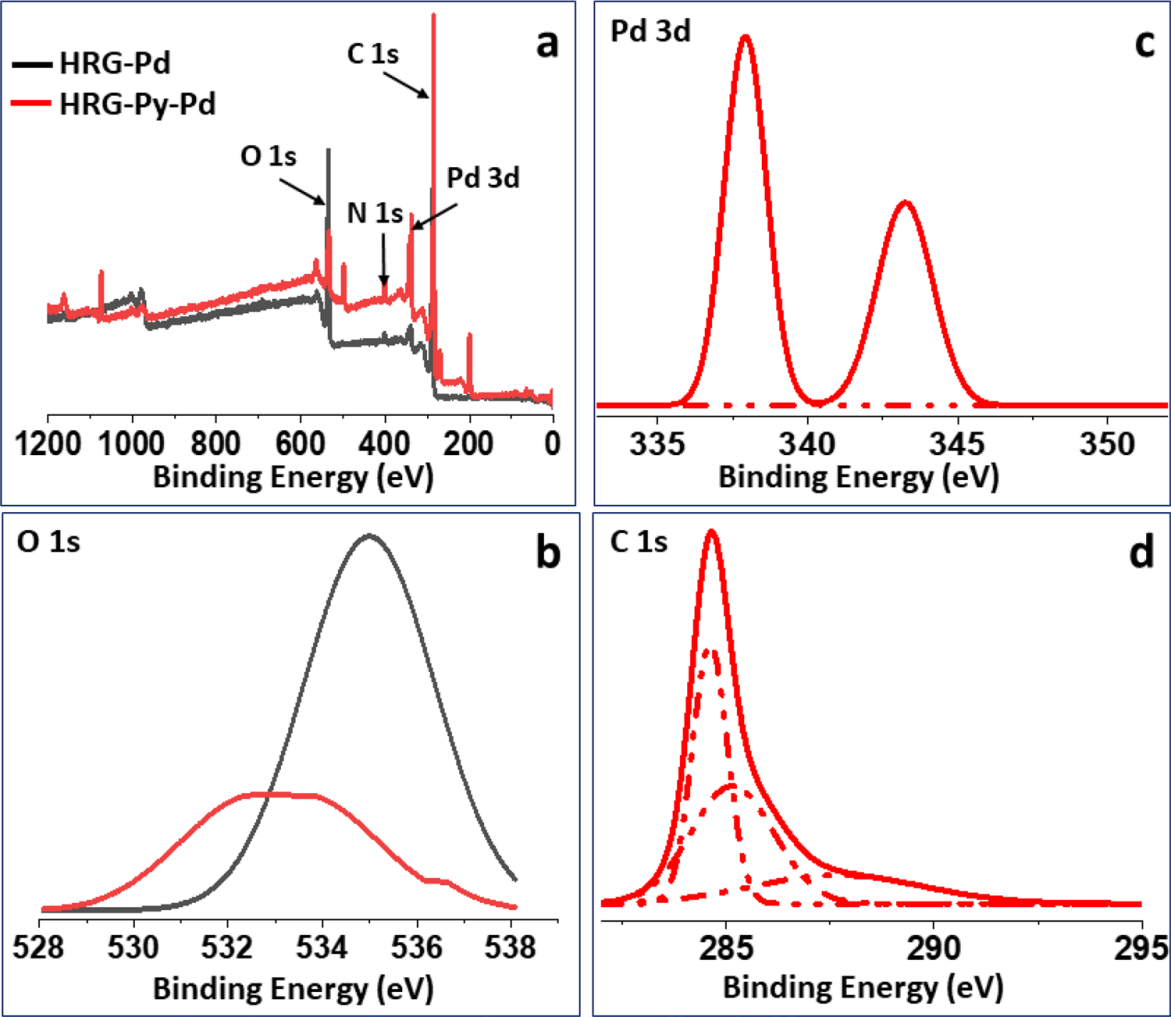
XPS analysis of HRG-Pd and HRG-Py-Pd (**a**) Comparative survey scan of HRD-Pd and HRG-Py-Pd (**b**) Comparative high resolution XPS analysis of O 1s spectrum for palladium nanoparticles (**c**) High resolution XPS analysis of Pd 3d spectrum for palladium nanoparticles (**d**) High resolution XPS analysis of C 1s spectrum for HRG-Py-Pd composites.

The XPS survey scan reveals the presence of C and O and N. The O1s peak could be due to the oxygen containing functional groups present on the surface of HRG. The C 1s and Pd 3d peaks are due to graphene and palladium. However, in the case of HRG-Py-Pd, an additional signal corresponding to that of N 1s was obtained which could be attributed to the ‘N’ from 1-AP. Comparisons between the high resolution scan of the O 1s, Pd 3d, and C 1s was also carried out and is presented in the Fig. 6 (b), (c) and (d) respectively. The high resolution spectrum of O 1s gives a signal with binding energy maximum at 535 eV for the HRG-Pd while the HRG-Py-Pd yields a signal at 533 eV, moreover the peak area relevant to O 1s in the HRG-Pd signal was found to be two times broader than the HRG-Py-Pd which indicates that incorporation of 1-AP on the surface of HRG-Pd, decreases the surface oxygen functionalities. Similarly differences were found in the case of the high resolution scan of Pd 3d and C 1 s. The high resolution scan of Pd 3d for the HRG-Pd yielded signals at 338.5 eV and 344.5 eV with shoulders at 343.8 and 346.1 eV, indicating the presence of some Pd^2+^. This could be due to the interaction of Pd precursor with surface oxygen moieties present on the graphene or due to surface oxidation of Pd. However, the HRG-Py-Pd gave sharp peaks for Pd centered at 337.9 eV, 343.2 eV respectively, indicating the presence of only Pd^0^. The difference of binding energy being 5.3 eV is indicative of existence of Pd 3d_5/2_ and Pd 3d_3/2_ of Pd(0) in the HRG-Pd and HRG-Py-Pd composite^40^. Upon examination of the reused catalyst using XPS, it was found that there is no change in the intensity and oxidation state of the Pd nanoparticles deposited on the HRG-Py-Pd. (Fig. S2) Furthermore, the high resolution scan of C 1s spectrum for the HRG-Pd composite revealed signals at 287 eV and 289.5 eV, while the HRG-Py-Pd composite yielded signals at 284.6 eV, 285 eV and 288 eV. The peaks at 287 eV and 289.5 eV can be attributed to –C = O and –C–OH. The most intense peak at 284.6 eV and 285 eV corresponds to carbon in the –C–C, –C–H or –C = C bonds, while the peak at 288 eV, can be attributed to –C–NH_2_ which arises due to the incorporation of 1-AP on the surface of HRG-Pd^41–44^.

### TEM and EDX analysis

**Figure 7:**
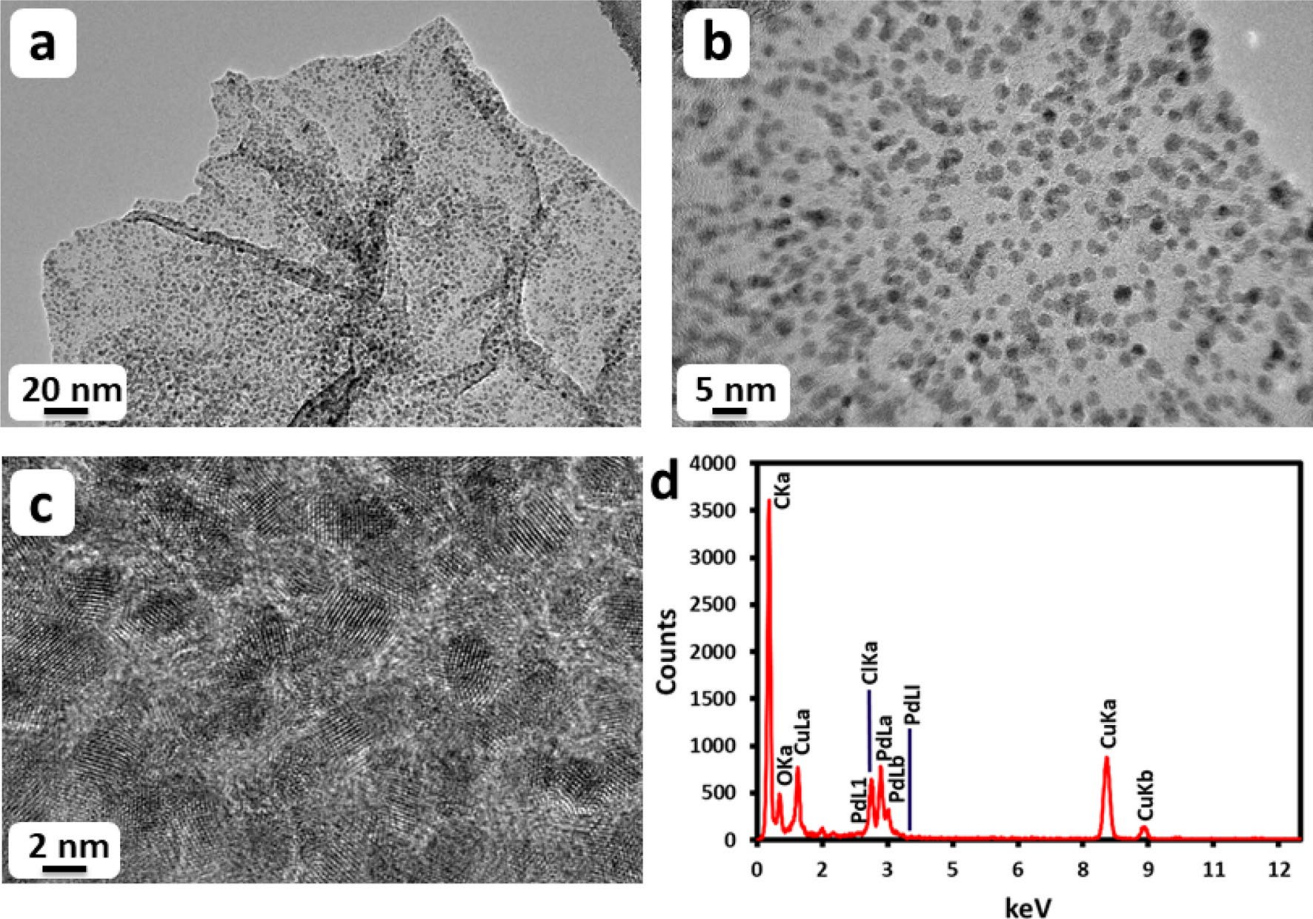
TEM images of HRG-Py-Pd; (**a**, **b**) overview images showing the homogeneity and monodispersity of Pd nanoparticles, (**c**) HRTEM image confirming the monocrystalline nature of Pd nanoparticles and (**d**) EDX spectrum indicating the presence of C and Pd.

Morphology and the size of Pd NPs on the surface of HRG both in HRG-Py-Pd and HRG-Pd was analyzed using high resolution transmission electron microscopy (HRTEM). The Fig. 7 presents the HRTEM data for HRG-Py-Pd nanocomposites where the TEM images of HRG-Pd are shown in supplementary materials (Fig. S3). Notably, HRG-Py-Pd, due to the presence of 1-AP exhibit dense and homogeneous dispersion of ultrafine smaller size Pd NPs on the surface of HRG (Fig. 7), whereas, the HRG-Pd shows larger size excessively aggregated Pd NPs (Fig. S3a). The EDX spectrum of the HRG-py-Pd nanocomposite synthesized through functionalization (Fig. 7d) shows relatively higher amount of Pd on comparing the Pd : C ratio with that of Pd : C obtained from the HRG-Pd nanocomposite (Fig. S3b), synthesized without functionalization. The histogram showing the size of resulting nanoparticles for both HRG-Py-Pd and HRG-Pd are given in the supplementary materials Fig. S4. Since, 1-AP provided suitable surface chemistry and effective active sites for the nucleation and growth of homogeneous size Pd NPs. Additionally, it was also observed that, due to the superior stabilization of HRG through π–π interactions between pyrenyl ring of 1-AP and the basal plane of HRG, the aggregation of HRG nanosheets is largely prevented. This resulted in the enhancement of the surface area, which provides more active sites for the higher loading of Pd NPs and a significantly enhanced catalytic activity.

### Catalytic application

Among various catalytic transformations, the Suzuki–Miyaura coupling is one of the most extensively studied organic reactions, which has vast industrial applications^23^. Pd nanoparticles and other Pd based catalysts are the most common choice for the Suzuki–Miyaura couplings, due to their superior stability and excellent catalytic activities^45,46^. Such types of coupling reactions are usually carried out at higher temperatures in various organic solvents, such as THF^47^. However, the organic wastes and toxic gases generate during these reactions causes adverse effects on the environment and higher temperatures require more energy, which increases the cost^48^. Apart from Pd based heterogeneous catalysts, the Suzuki couplings are also carried out using Pd complexes based homogeneous catalysts^49^. But, these systems need activation by phosphine ligands, which are air sensitive and require inert conditions. Moreover, homogeneous catalysts are not easy to separate and usually suffer from reusability problems, which inhibit their large scale industrial applications^50^.

Therefore, developing Pd based heterogeneous catalysts which do not require activation by phosphine ligands and can be use under aqueous conditions is highly desirable. Significant efforts have been carried out to develop several Pd NPs based heterogeneous catalysts for the Suzuki coupling reactions under water^51^. Notably, various organo boronic acids, which are commonly applied during the Suzuki coupling reactions in aqueous conditions, effectively tolerate the presence of water and a variety of functional groups^52^. Pd based heterogeneous catalysts for the Suzuki coupling reactions under aerobic conditions have attracted tremendous attention^51^. However, a lot of work needs to be done to overcome several challenges in this regard^53^. For instance, the efficiency and reusability of Pd catalysts is severely hampered due to the aggregation of NPs^54^. It is reported in the literature that the aggregation of Pd NPs can be effectively controlled by the proper dispersion of NPs on the surface of efficient support materials, such as, metal oxides, inorganic porous and carbon materials^55^.

**Scheme 2:**
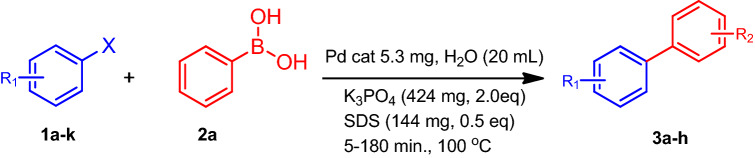
HRG‐Py-Pd and HRG‐Pd nanocatlyst catalyzed Suzuki coupling reaction with different aryl halides having neutral, + *I* and *–I* effects in nature.

The HRG-Py-Pd catalyst described above was applied to the Suzuki–Miyaura coupling reactions, which neither require any pre-activation nor working under inert conditions. The catalytic activity of the 1-AP functionalized HRG-Py-Pd is compared with non-functionalized HRG-Pd catalyst. These catalysts were applied for the catalytic coupling of substituted aryl halides containing different types electron donating groups (EDG) i.e. + *I* or electron withdrawing groups (EWG) i.e.* −I*, such as, chloro, bromo and iodobenzene, 4-chlorobenzophenone and 4-Bromoanisol etc., with a variety of phenylboronic acids to produce biphenyls (cf. Scheme 2). The reactions were performed in water containing sodium lauryl sulfate and K_3_PO_4_ under aerobic conditions. Due to the effective stabilization of HRG-Py-Pd catalyst by 1-AP, it can be easily dispersed in the solvent (water) with simple stirring and can be separated from the reaction mixture using centrifugation. Whereas, the non-functionalized HRG-Pd catalyst exhibited poor dispersibility in water, due to which its catalytic activity was considerably affected.

As stated earlier, the functionalization approach not only provides the functional groups for the binding of Pd nanoparticles but also avoids agglomeration that in turn increases the surface area. This was further confirmed by measuring the specific surface area of the as-prepared HRG-Py-Pd and HRG-Pd samples. The samples were measured using BET nitrogen adsorption after degassing at 120 °C for 16 h. It was revealed that the HRG-Py-Pd exhibited higher surface of 553.44 m^2^ g^−1^ when compared to the surface area of HRG-Pd (472.71 m^2^/g). Additionally, turnover frequency (TOF) values for the both nanocatalysts (HRG-Py-Pd and HRG@Pd) were calculated, the data is presented in the supporting information (Fig. S5). The TOF value for the HRG-Py-Pd is 5 folds greater than that of HRG@Pd. This enhancement in catalytic activity can be attributed to the better catalyst as result of functionalization with 1-aminopyrene. This led to the better control on size and dispersion of Pd nanoparticles on the surface of the HRG leading to enhanced catalytic activity i.e. 100% coupled product within 5 min of (Iodobenzene precursor) reaction time. The product (HRG-Pd) obtained with functionalizing the HRG, took 20 min to perform the same coupling reaction. It was found that the TON values of HRG-Py-Pd are almost similar to HRG-Pd and the previously reported nitrogen doped graphene@palladium (NDG@Pd) composite (reference is provided in the Supp Info). However, regarding the TOF values, HRG-Py-Pd was found to be 5 folds better than HRG-Pd, indicating the important role of 1-AP, in enhancing the catalytic performance. Notably, the enhanced surface area of HRG-Py-Pd is attributed to the superior stabilization of the HRG and homogeneous distribution of Pd NPs on the surface of HRG.

**Figure 8:**
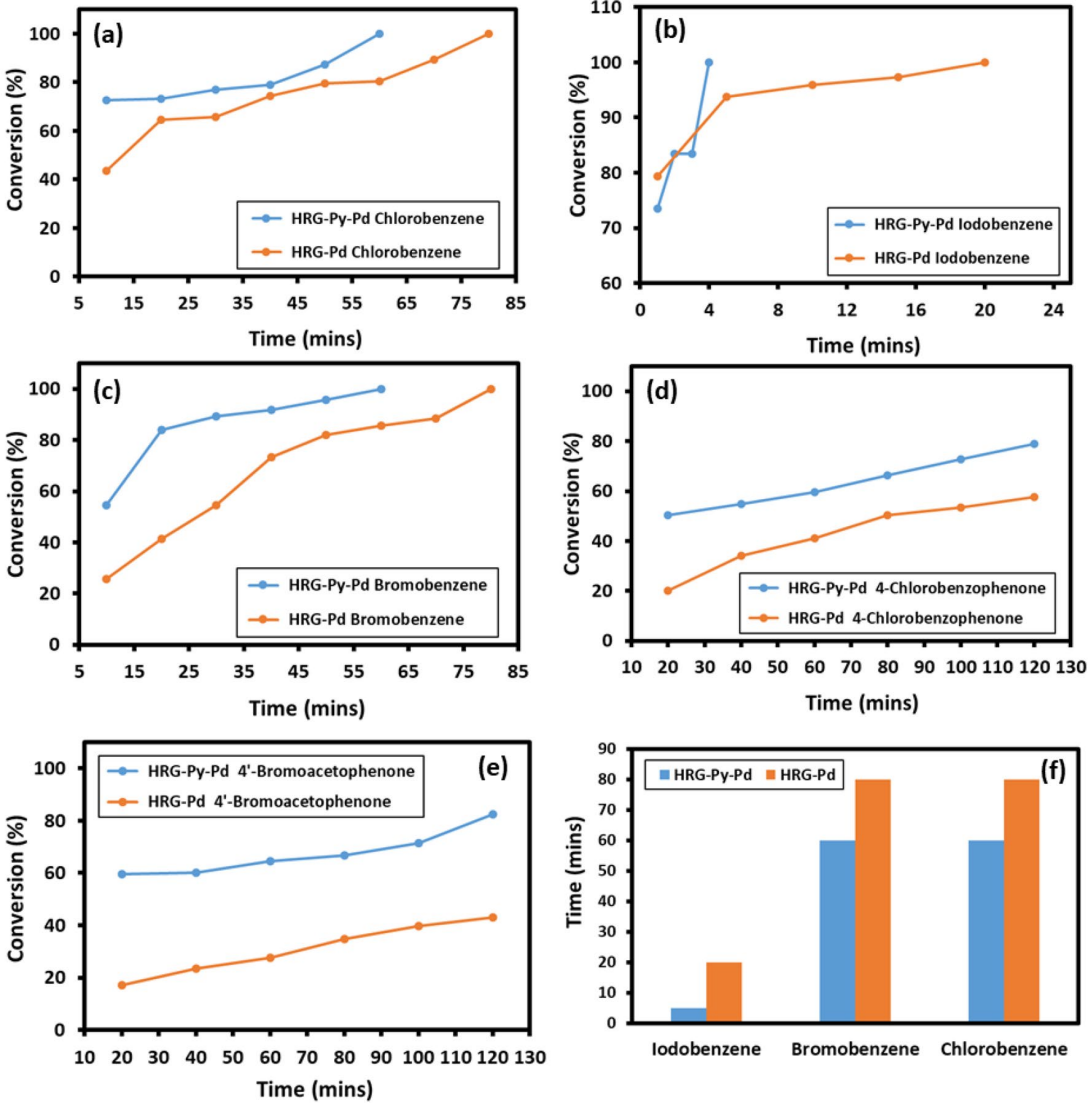
Time dependent conversion efficiency of the Suzuki reaction employing HRG‐Py-Pd and HRG‐Pd for various substrates using GC analysis: (**a**) chlorobenzene, (**b**) iodobenzene and (**c**) bromobenzene, (**d**) 4-chlorobenzophenone, (**e**) 4′-bromoacetophenone (**f**) comparison of the conversion (100%) of product using HRG-Py-Pd and HRG-Pd with respect to time.

The detailed catalytic evaluation of the as-prepared catalysts **(**HRG-Py-Pd and/or HRG-Pd) for the Suzuki coupling reactions of some of the aryl halides is compiled in Fig. 8. The results were compared with the catalyst obtained without pyrene functionalization **(**HRG-Pd). The (HRG-Py-Pd) nanocatalyst was evaluated for the Suzuki–Miyaura coupling of chloro, bromo and iodo-benzene and different types of substituted aryl halides containing both EDG and EWG with a variety of phenylboronic acids in water. Initially, to test the importance of Pd based catalysts in the coupling reactions, a blank reaction is performed using iodo benzene and phenyl boronic acid as substrates under same conditions. No product is formed in the absence of catalyst. Therefore, the reactions were repeated using both HRG-Py-Pd and HRG-Pd catalysts. It was found that the coupling product formation using the iodo substituted benzene requires the least reaction time and yields a complete conversion product within ~ 5 min of reaction when the catalyst (HRG-Py-Pd) is employed, while the same conversion product took 20 min using HRG-Pd as catalyst. Similar pattern of catalytic performance was observed when the bromo, chloro—substituted benzene were subjected to coupling reaction. Apart from these compounds, the catalytic efficiency of HRG-Py-Pd has also been tested for various substituted aryl halides including 4-chlorobenzophenone, 4′-bromoacetophenone, and 4-Bromoanisol. The substituted aryl halides have demonstrated slightly less conversion when compared with non-substituted aryl halides, due to the steric hindrance caused by the substituents. Moreover, among various substituted aryl halides, compounds containing EDG have yielded higher conversion when compared with the aryl halides containing EWG. For instance, 4-chlorobenzophenone containing EWG as substituent has demonstrated lower conversion (78.9%) when compared with the 4-Bromoanisol (100%) which consists of EDG methoxy group. Furthermore, also in the case of substituted aryl halides, the HRG-Py-Pd has exhibited superior catalytic activities than the non-functionalized HRG-Pd. The conversions obtained are compiled in Table 1 and graphical representation of the results of some of the aryl halides is presented in the Fig. 8.

**Table 1:** Time dependent conversion efficiency of the Suzuki reaction employing HRG‐Py-Pd and HRG‐Pd for different aryl halides including iodobenzene, bromobenzene, chlorobenzene, 4-bromoanisol, 4-bromoacetophenone etc.

+ / − Groups	Aryl halides	Product ID	HRG-Py-Pd		HRG-Pd	
			Time (mins)	Conversion (%)	Time (mins)	Conversion (%)
Neutral	Chlorobenzene (**1a**)	**3a**	60	100	80	100
	Bromobenzene (**1b**)	**3a**	60	100	80	100
	Iodobenzene (**1c**)	**3a**	5	100	20	100
EWG	4-Chlorobenzophenone (**1d**)	**3b**	120	78.9	120	57.7
	4-Bromoacetophenone (**1e**)	**3c**	120	82.3	120	42.9
	4-Chlorobenzoic acid (**1f**)	**3d**	120	15	120	12
	4-Bromobenze-sulfonylchloride (**1g**)	**3e**	60	97	20	93
EDG	4-Bromoanisol (**1h**)	**3f**	60	100	20	100
	2-Bromoaniline (**1i**)	**3g**	60	97	20	90
	2-Iodoaniline (**1j**)	**3g**	60	100	60	95
	4-Iodotoluene (**1k**)	**3h**	20	100	20	100

**Reaction Condition**: Aryl halide (1 mmol), boronic acid (1.2 mmol), SDS (0.5 mmol), K_3_PO_4_ (2 mmol), Water (20 mL); Note: EWG = Electron withdrawing groups; EDG = Electron donating groups.

**Scheme 3:**
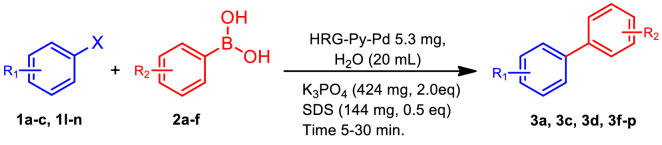
HRG‐Py-Pd catalyzed Suzuki coupling reaction of with aryl halides and boronic acids bearing different subtituents.

Once the superior catalytic activity of HRG-Py-Pd is established, the effect of amount of Pd on the catalytic activity of the nanocatalyst is studied by preparing two different samples of HRG-Py-Pd by varying the Pd contents. For this purpose, two samples were prepared using 0.05 wt.% Pd precursor (*HRG-Py-Pd) and 0.5 wt% Pd precursor (**HRG-Py-Pd) while using the same amount of HRG. The catalytic activity of HRG-Py-Pd is linearly decreased with the amount of Pd. For example, only 42 and 62% of conversion was obtained in case of iodobenzene when *HRG-Py-Pd and **HRG-Py-Pd were used respectively under similar set of reaction conditions when compared with HRG-Py-Pd (50 wt.%). More details and the results of other coupling reactions using catalysts with low Pd contents are described in Table 2. We have also performed the catalytic activity of the HRG-Py-Pd catalyst using substituted boronic acid as substrates as described in Scheme 3. The effect of substituents in case of substituted boronic acid was negligible on the catalytic activity of HRG-Py-Pd, and the catalyst remained active in the presence of all the boronic acid used in this study. Among different boronic acids, in this case phenyl boronic acid proved as the most efficient substrate for Suzuki coupling reactions using HRG-Py-Pd as catalyst (cf. Table 3).

**Table 2:** Time dependent conversion efficiency of the Suzuki coupling reaction employing HRG‐Py-Pd with lower contents of Pd where * = HRG-Py-Pd 0.05 wt% and ** = HRG-Py-Pd 0.5 wt.%. All the reactions were performed using phenyl boronic acid.

S. no	Aryl halides	Product	Time (min.)	GC conversion (%)
1	Chlorobenzene (**1a**)*	**3a**	6 h	10
2	Chlorobenzene (**1a**)**	**3a**	6 h	12
3	Bromobenzene (**1b**) *	**3a**	6 h	43
4	Bromobenzene (**1a**) **	**3a**	6 h	49
5	Iodobenzene (**1c**) *	**3a**	6 h	46
6	Iodobenzene (**1c**) **	**3a**	6 h	62

**Table 3:** Suzuki coupling reaction of different aryl halides with aryl boronic acid bearing different substituents in the presence of HRG‐Py-Pd nanocatalyst.

S. no	Aryl halides	Boronic acids (R_2_)	Product	Time (min.)	GC conversion (%)	Isolated yield (%)
1.	Iodobenzene (**1c**)	4-COCH_3_ (**2b**)	**3c**	60	93	90
2.	Iodobenzene (**1c**)	4-COOH (**2c**)	**3d**	60	97	95
3.	Iodobenzene (**1c**)	4-OCH_3_ (**2d**)	**3f**	30	100	98
4.	Iodobenzene (**1c**)	4-CF_3_ (**2e**)	**3i**	30	100	97
5.	2-methoxy- bromobenzene (**1l**)	4-F (**2f**)	**3j**	80	93	91
6.	2-methoxy- bromobenzene (**1l**)	4-COCH_3_ (**2b**)	**3k**	80	92	89
7.	3-methoxy- bromobenzene (**1m**)	4-F (**2f**)	**3l**	60	100	98
8.	3-methoxy- bromobenzene (**1m**)	4-COCH_3_ (**2b**)	**3m**	80	97	92
9.	3-methoxy- bromobenzene (**1m**)	4-CF_3_ (**2e**)	**3n**	60	100	98
10.	3-methoxy- bromobenzene (**1m**)	4-OCH_3_ (**2b**)	**3o**	80	99	96
11.	2-Bromo-Pyridine (**1n**)	4-COCH_3_ (**2b**)	**3p**	120	65	59

**Reaction Condition**: Aryl halide (1 mmol), boronic acid (1.2 mmol), SDS (0.5 mmol), K_3_PO_4_ (2 mmol), Water (20 mL). Note: Isolated yields: after purification through a small pad of silica and Celite.

The higher catalytic activity of HRG-Py-Pd catalyst for the coupling reactions was attributed to its high surface area (553.44 m^2^ g^−1^) in comparison with HRG-Pd catalyst (472.71 m^2^/g). The surface area of the former catalyst is enhanced due to the higher loading and homogeneous distribution of the Pd NPs. Although, same amount of Pd precursor (Sodium tetrachloropalladate) was used during the preparation of both HRG-Py-Pd and HRG-Pd, however, the HRG-Py-Pd has demonstrated enhanced loading and homogeneous distribution of Pd NPs on the surface of HRG, due to the presence of pyrene. Pyrene has not only stabilized the surface of HRG but it also helped in the firm anchoring of Pd NP. Whereas, in the case of HRG-Pd, the Pd NPs not only aggregated but also failed to stick to the surface of HRG leading to the lower content of active Pd in the resultant catalyst. This has resulted in the lower catalytic activity of the HRG-Pd. Similarly, the lower catalytic activities of the non-functionalized HRG-Pd when compared to the functionalized catalyst have also been reported in earlier studies. For instance, graphene-Pd catalyst reported by Li et al., has exhibited lower conversions of less than 100% in the time range of 10–120 min for the conventional aryl halides including chloro, bromo and iodo benzene^56^. From the results obtained it can be concluded that functionalization of HRG with pyrene has positive impact both to avoid agglomeration of graphene sheets as well as nucleation and growth of monodisperse and monocrystalline Pd nanoparticles. These factors lead to the improved catalytic performance towards Suzuki–Miyaura coupling reaction.

The catalyst reusability is an essential parameter which defines the commercial significance of the material. To evaluate the reusability and the stability of HRG-Py-Pd, the couplings of iodo benzene and chlorobenzene were selected as model reactions. Initially, after performing the coupling reaction with freshly prepared HRG-Py-Pd, the catalyst was recovered from the reaction mixture by centrifugation. The collected material was washed several times with DI water (which is also used as solvent in repeated reactions) and dried at 100 °C for several hours to avoid contamination with reactant or product of the previous reactions. The recovered catalyst was reused for up to 5 times in a similar manner to test the stability and quality of the catalyst and final product of the coupling reaction. The freshly prepared HRG-Py-Pd has yielded 100% conversion in the case of both chlorobenzene and iodo benzene, and has also demonstrated almost similar catalytic activity and selectivity when reused for several times with slightly reduced conversion (up to 5 to 6% in case of both chloro and iodo benzene) as shown in Fig. S6 in the supplementary information. The structural stability of the reused catalyst was confirmed by further characterization using XRD and TEM (the data is provided in the Fig. S7 and Fig. S8 of supporting materials). The amount of Pd has also remained intact on the surface of HRG, which is confirmed by XPS analysis.

## Experimental details

### Materials required

Graphite powder (99.999%, −200 mesh) was purchased from Alfa Aesar. All other materials and organic solvents were purchased from Sigma-Aldrich and used without further purification. Materials used are 1-aminopyrene (97%), sodium tetrachloropalladate (II) (99.9%), concentrated sulfuric acid (H_2_SO_4_) (98%), potassium permanganate (KMnO_4_) (99%), sodium nitrate (NaNO_3_) (99%), hydrogen peroxide (H_2_O_2_) (30 wt.%), bromobenzene (99.5%), chlorobenzene (99%), iodobenzene (99%), 4-chlorobenzophenone (99%), 4-bromoacetophenone, 4-chlorobenzoic acid, 4-bromobenze-sulfonylchloride, 4-bromoanisol, 2-bromoaniline, 2-iodoaniline, 4-iodotoluene, sodium dodecyl sulfate (98%), phenyl boronic acid (95%), tripotassium phosphate (98%).

### Preparation of graphene oxide (GRO)

Graphite oxide (GO) was synthesized from graphite powder using a modified Hummers method^57,58^. Briefly, graphite powder (0.5 g) and NaNO_3_ (0.5 g) were taken in 23 ml of H_2_SO_4_. The mixture was allowed to stir for several minutes (~ 10 min) in an ice bath. Subsequently, KMnO_4_ (3 g) was slowly added (the color of the mixture turned to dark green) to this mixture. After proper mixing, the ice bath is replaced with water bath, which is maintained at temperature between 35 and 40 ºC for one hour, resulting in the formation of a thick paste. Thereafter, 40 ml of water was added, and the mixture was stirred for another 30 min at a temperature of ~ 90 ºC. Finally, 100 ml of water was added, which is followed by the slow addition of 3 ml of H_2_O_2_. This resulted in the color change of the mixture from dark brown to yellowish color. The mixture was allowed to cooled, and subsequently filtered and washed with 100 ml of water. The resulting thick brown paste was dispersed in water (100 ml) and centrifuged at a low speed of 1,000 rpm for 2 min. This step was repeated for several times (4–5 times), until all unsettled particles were removed. Then, the same step is repeated at a high speed of centrifugation at 8,000 rpm to remove remaining small pieces of GRO. After this, the resultant paste is redispersed in water via mild sonication to obtain a solution of GRO.

### Reduction of highly reduced graphene oxide (HRG)

GRO is reduced according to a previously reported method^59^. Briefly, 100 mg of GRO is dispersed in water (30 ml) and sonicated for 30 min. The resulting suspension was allowed to heat up to 100 ºC, and subsequently 3 ml of hydrazine hydrated was added. After sometime, the temperature was slightly reduced (98 ºC), and the suspension was kept under stirring for 24 h. Finally, a black powder is obtained which is filtered and washed several time with water to remove excessive hydrazine. In order to remove remaining bulk graphite, the resultant suspension is centrifuged at slow speed (4,000 rpm) for several minutes (3–4 min), and the final product is collected via filtration and dried under vacuum.

### Functionalization of HRG with 1-aminopyrene

25 mg of HRG was dispersed in 10 ml of methanol via sonication for 30 min. This dispersion is added to the solution of 25 mg of 1-Aminopyrene in methanol (10 ml). The mixture was stirred for 48 h at room temperature and then sonicated for 6 h at 20 ºC. Then the mixture was centrifuged 3 h to remove the excess of aminopyrene. Further purification was carried out to remove un-adsorbed aminopyrene. For this purpose, the black mixture is redispersed in 5 mL of fresh methanol and sonicated for 30 min at 20 °C, subsequently; the black suspension is centrifuged for 1 h, and the product is isolated by decanting the resulting mixture. This process was repeated (at least three times) until the solution in the centrifuge tube turned colorless. The product was dried under vacuum overnight.

### Preparation of functionalized graphene and palladium composites (HRG-Py-Pd)

In order to prepare the graphene-palladium nanocomposites (1:1 wt eq), the 5 ml dispersion of aminopyrene functionalized HRG in ethanol (1 mg HRG/ml of ethanol) was added to 5 ml solution of Na_2_PdCl_4_ in ethanol (5 mg, 0.0169 mmol). The resultant mixture was sonicated for 1 h. The product was isolated by centrifugation (9,000 rpm) and redispersed in 10 ml of water for further use. The HRG-Pd was also prepared in the similar manner, except in this case, pristine HRG was used instead of functionalized HRG. The samples with low content of Pd are also prepared according to aforementioned method using 0.05 (*HRG-Py-Pd) and 0.5 wt.% (**HRG-Py-Pd) of Na_2_PdCl_4_ with respect to starting amount of HRG.

### Catalytic activity

The catalytic protocol was followed as earlier reported by us^18^. In a typical experiment, a mixture of sodium dodecyl sulfate (144 mg, 0.5 mmol), tripotassium phosphate (K_3_PO_4_, 399 mg), phenylboronic acid (146 mg, 1.2 mmol) and deionized water (20 mL) was taken in a 100 mL round bottom flask. Halobenzene (1.0 mmol) was added to this mixture under stirring, followed by the as-prepared HRG-Py-Pd and/or HRG-Pd nanocatalyst (5 mol.%, 5.32 mg). The mixture was stirred at 100 ºC in an oil bath for 5 min and then extracted with ethyl acetate (3 × 20 mL). The combined organic extract was dried over anhydrous sodium sulfate (Na_2_SO_4_), and the resulting mixture was analyzed by gas chromatography (GC). In order to identify the product obtained from the catalytic reaction, the as-obtained mixture was crystallized from ethanol. The resulting product was characterized using ^1^H and ^13^C solution NMR and mass spectroscopy^18^. M.p.: 68–70 °C (69–71 °C Ref. Supp Info reference 1); ^1^H NMR (400 MHz, CHLOROFORM-*D*) δ 7.49 (d, *J* = 7.7 Hz, 4H, Ar–H), 7.33 (t, *J* = 7.7 Hz, 4H, Ar–H), 7.23 (t, *J* = 7.3 Hz, 2H, Ar–H); ^13^C NMR (101 MHz, CHLOROFORM-*D*) δ 141.34, 128.99, 128.75, 127.36, 127.18; electron impact-mass spectrometry (EIMS) m/z 154 (M +). The ^1^H and ^13^C solution NMR spectra of all other products obtained during this study and the details of their spectra anaylsis are provided in the supplementary information (Figs. S9–S38).

### Characterization

The as-synthesized HRG-Py-Pd and/or HRG-Pd nanocatalysts and the product obtained from the Suzuki reactions were characterized by UV–Vis spectroscopy (Perkin Elmer lambda 35 (Waltham, MA, USA)), high resolution transmission electron microscopy (HRTEM) and EDX (JEM 2100F (JEOL, Tokyo, Japan)), FT-IR spectroscopy (Perkin Elmer 1,000 FT-IR spectrometer)^60^, (Agilent spectrometer (single quadrupole) MSD-5975C detector, Agilent Technologies Inc., USA, MS was acquired in EI mode (scan range m/z 45–600, ionization energy 70 eV)). Gas chromatography (GC) (GC 7890A, Agilent Technologies Inc., equipped with a flame ionization detector (FID) and a 19019S-001 HPPONA column)^21^. The XRD analysis of the as-prepared nanocatalysts were carried out using a D2 Phaser X-ray diffractometer (Bruker, Germany), Cu Ka radiation (k = 1.5418 A°). XPS spectra were measured on a PHI 5,600 Multi-Technique XPS (Physical Electronics, Lake Drive East, Chanhassen, MN) using monochromatized Al Ka at 1,486.6 eV. Peak fitting was performed using the CASA XPS Version 2.3.14 software^18^. Flash chromatography was performed on 100–200 mesh silica gel. ^1^H and ^13^C Nuclear Magnetic Resonance (NMR) spectra were recorded on JEOL-400 MHz spectrometers at ambient temperature in CDCl_3_ & DMSO-d_6_ which were purchased from Sigma Aldrich. Chemical shifts (ppm) are referenced to the residual solvent peak. Coupling constants, J, are given in hertz^18^. Abbreviations used in the designation of the signals: s = singlet, d = doublet, dd = doublet of doublets, ddd = doublet of doublet of doublets, dt = doublet of triplets, t = triplet, td = triplet of doublets, m = multiplet. Melting points were performed at London Metropolitan University.

## Conclusions

We have demonstrated a simple and efficient method for the preparation of graphene-Pd nanocomposite through surface functionalization. For this purpose, 1-AP was utilized to tailor the surface of graphene for the efficient loading of Pd NPs to prepare HRG-Py-Pd nanocomposite. 1-AP provided excellent active sites for the efficient growth and homogeneous dispersion of ultrafine Pd NPs on the surface of HRG. HRG-Py-Pd elucidated excellent stability and dispersibility when compared with HRG-Pd, (prepared without using 1-AP). The dense and homogeneous distribution of Pd NPs in the HRG-Py-Pd nanocomposite leads to the significant enhancement of its surface area, in comparison with HRG-Pd. Unlike HRG-Pd, the HRG-Py-Pd demonstrated superior catalytic activities toward various Suzuki reactions, due to its enhanced properties. The catalytic conversions of different phenyl halides to biphenyl and other biphenyl derivatives, which were carried out under aerobic conditions, occurred in short time with less amount of Pd. Thus, the facile method presented here may provide an excellent opportunity for the preparation of other high quality graphene inorganic NPs based nanocomposites using different PAHs, including other pyrene derivatives as noncovalent functionalizing agents.

## Supplementary information

Supplementary information.
