# Supplementary material for: Facile synthesis of Pd@graphene nanocomposites with enhanced catalytic activity towards Suzuki coupling reaction
Source: Sci Rep. 2020 Jul 16;10:11728. doi: 10.1038/s41598-020-68124-w (PMC7366662; doi:10.1038/s41598-020-68124-w)
Supplement: Supplementary file 1 — Supplementary information. [file 41598_2020_68124_MOESM1_ESM.docx]

**Supplementary File**

**Facile Synthesis of Pd@Graphene Nanocomposites with Enhanced Catalytic Activity towards Suzuki Coupling Reaction**

**Mujeeb Khan^1,*^, Mohammed Rafi Shaik^1^, Syed Farooq Adil^1^, Mufsir Kuniyil^2^, Muhammad Ashraf^3^, Hajo Frerichs^4^, Massih Ahmad Sarif^4^, Mohammed Rafiq H. Siddiqui^1^, Abdulrahman Al–Warthan^1^, Joselito P. Labis^5^, Mohammad Shahidul Islam^1^, Wolfgang Tremel^4^_,_ and Muhammad Nawaz Tahir^3,*^**

^1^Department of Chemistry, College of Science, King Saud University, P.O. Box 2455, Riyadh 11451, Kingdom of Saudi Arabia

^2^Department of Chemistry, Koneru Lakshmaiah Education Foundation, Vaddeswaram,

Guntur 522502, Andhra Pradesh, India

^3^Department of Chemistry, King Fahd University of Petroleum and Minerals, P.O. Box 5048, Dhahran 31261, Kingdom of Saudi Arabia

^4^Institut für Anorganische Chemie und Analytische Chemie, Johannes Gutenberg-Universität, Duesbergweg 10-14, 55128 Mainz, Germany

^5^King Abdullah Institute for Nanotechnology, King Saud University, Riyadh 11451, Kingdom of Saudi Arabia

*Corresponding authors E–Mail: [kmujeeb@ksu.edu.sa, muhammad.tahir@kfupm.edu.sa](mailto:kmujeeb@ksu.edu.sa,%20muhammad.tahir@kfupm.edu.sa)


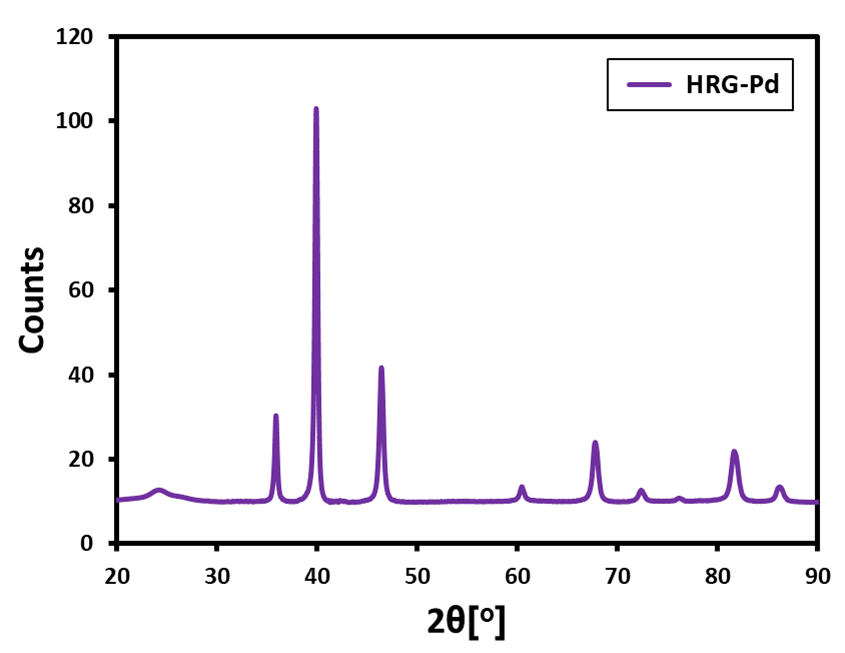


Fig. S1. XRD diffractograms of highly reduced graphene-palladium nanocomposite (HRG-Pd)

Fig. S2. High resolution XPS analysis of Pd 3d spectrum for (a) fresh HRG-Py-Pd (b) reused HRG-Py-Pd catalysts.


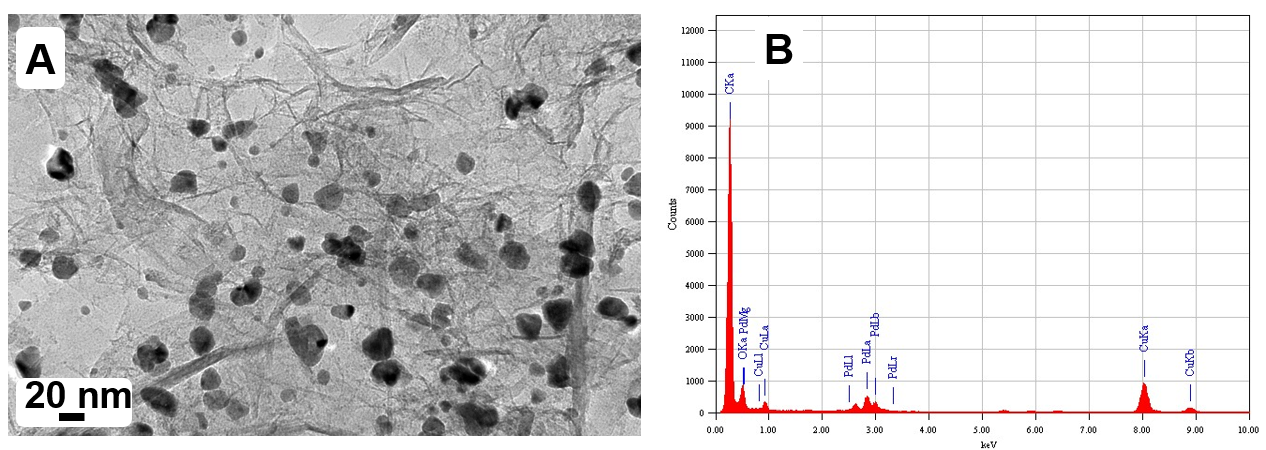


Fig. S3. (A) Transmission electron micrograph of as synthesized HRG-Pd (catalyst prepared without functionalizing the surface of HRG using 1-AP); (B) EDX spectrum indicating the presence and relative ratio of Pd and C in HRG-Pd sample.


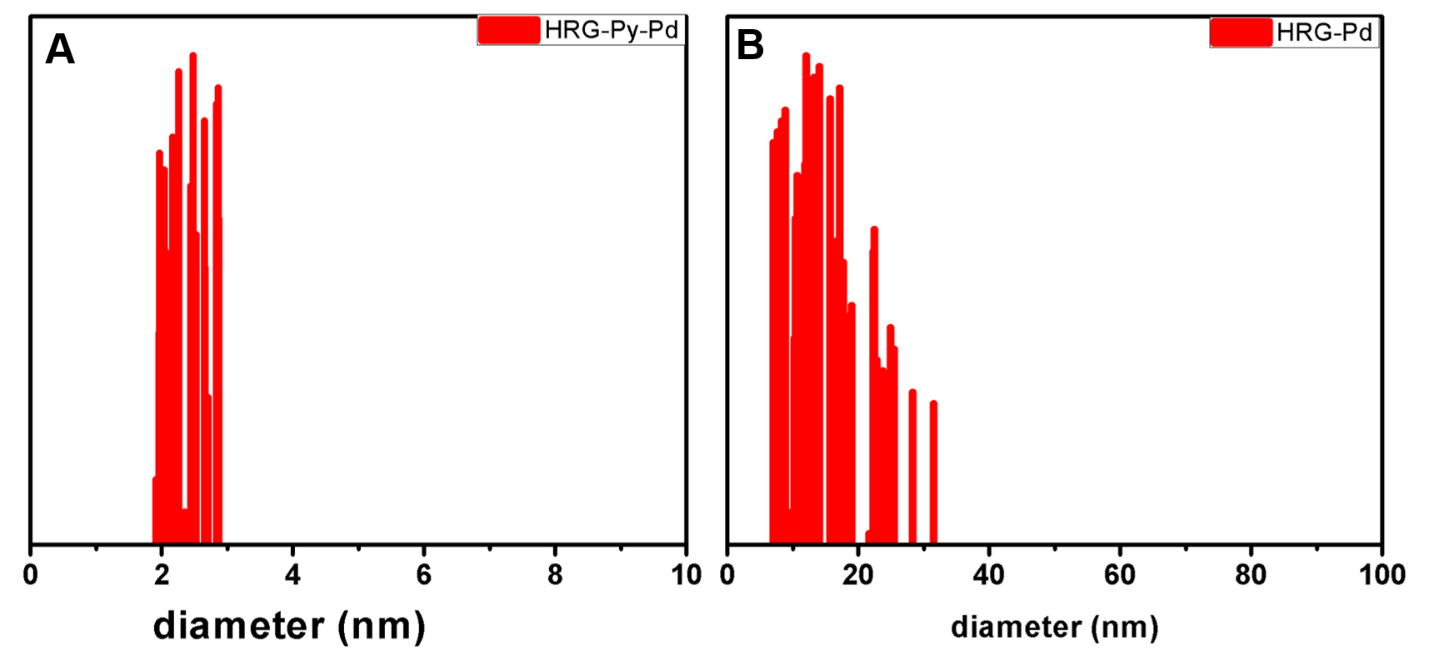


Fig. S4. The corresponding histograms indicating the particles size and size distribution; (A) HRG-Py-Pd and (B) HRG-Pd


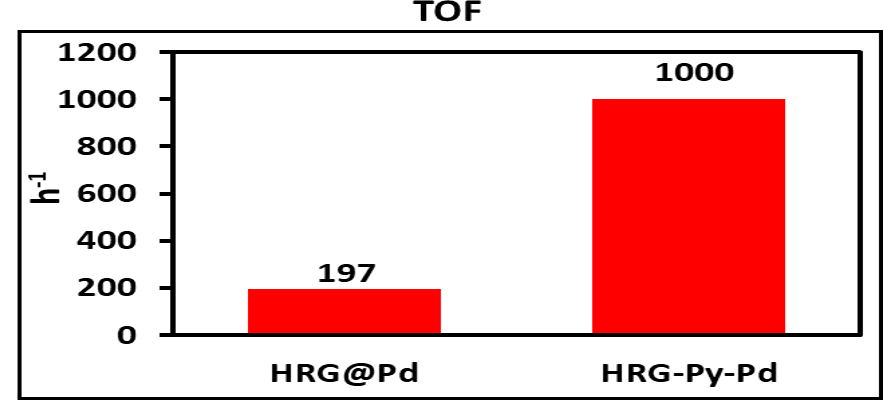


Fig. S5. TOF values of HRG-Py-Pd and HRG-Pd.


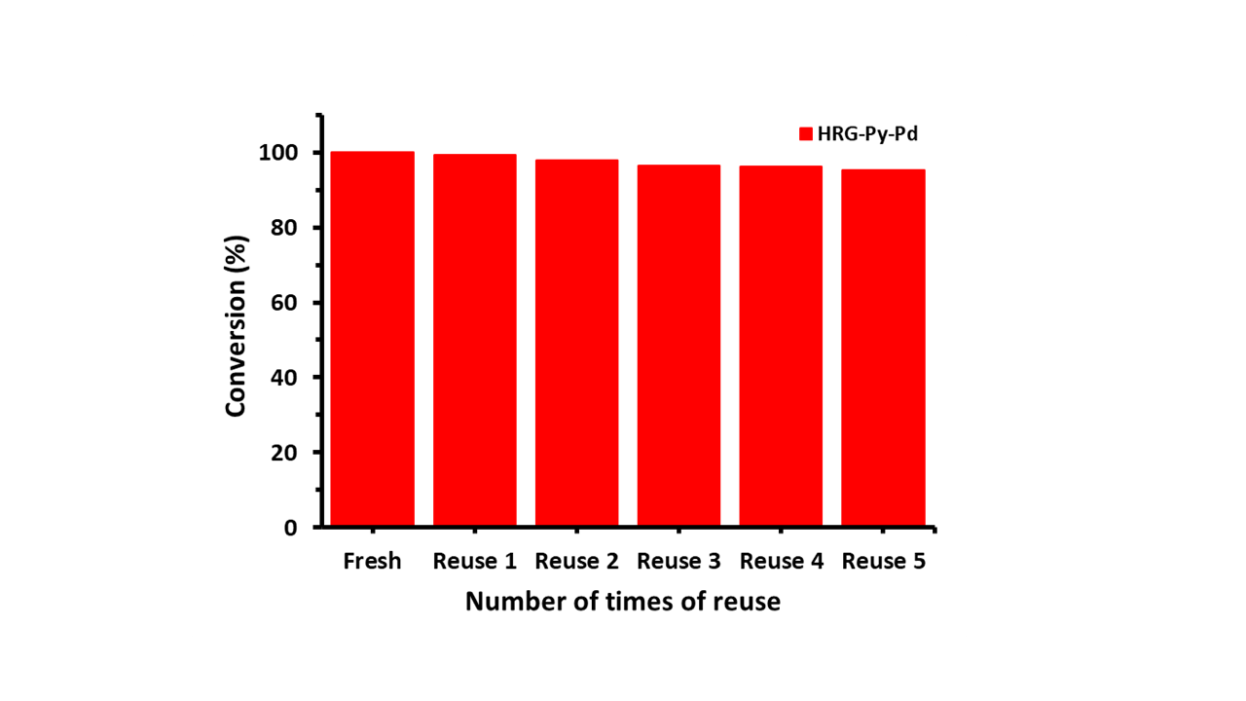


Fig. S6. Reusability study of HRG-Py-Pd used as a catalyst for the coupling of iodobenzene. Even after five cycles, the catalytic properties of HRG-Py-Pd remained intact.


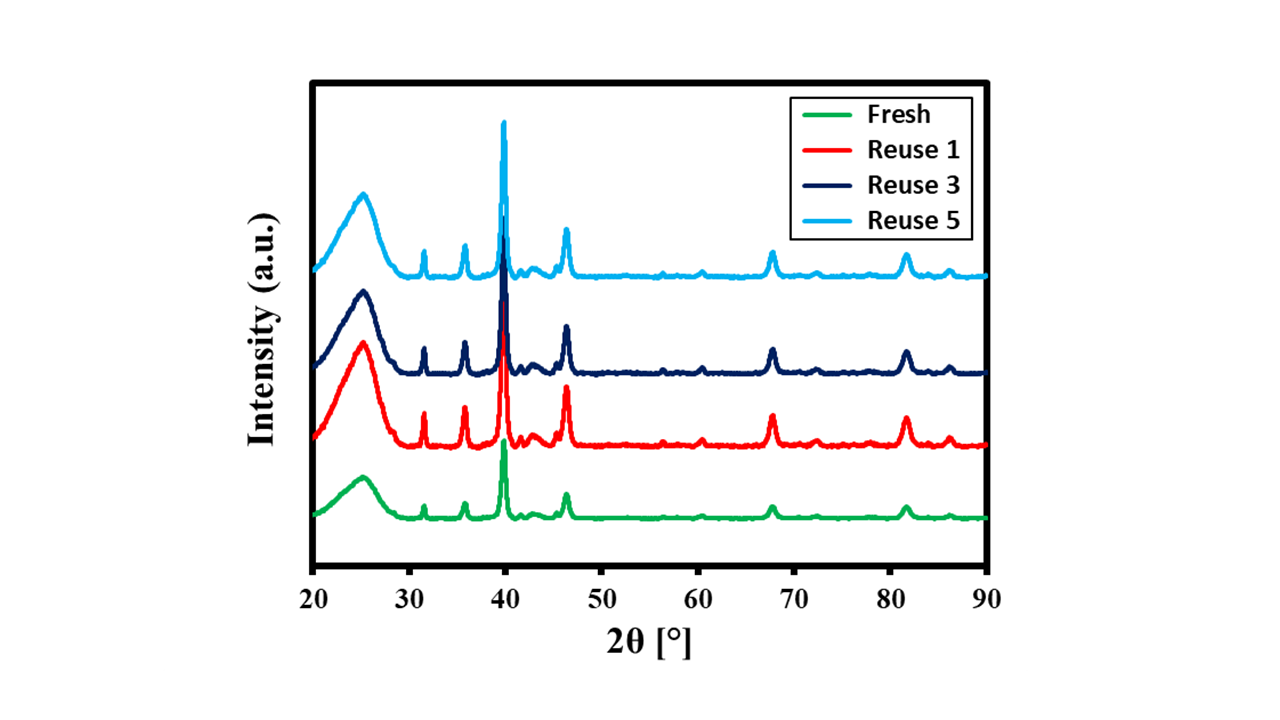


Fig. S7. XRD diffractograms of freshly prepared HRG-Py-Pd and after the reused catalysts during the coupling of iodobenzene.


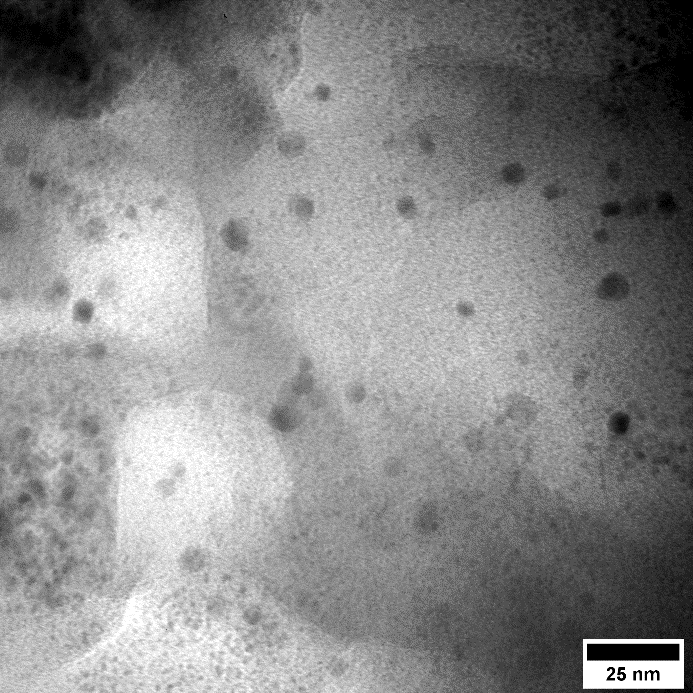

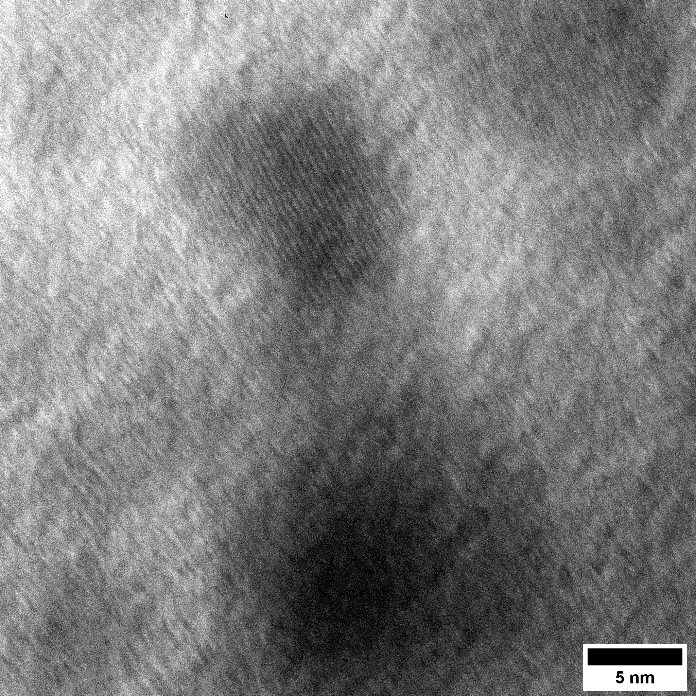


Fig. S8. TEM images of reused catalysts collected after 5 cycles during the coupling of iodobenzene; (A) overview TEM image showing very small size nanoparticles on HRG surface and (B) high resolution TEM (HRTEM) confirming the crystallinity.


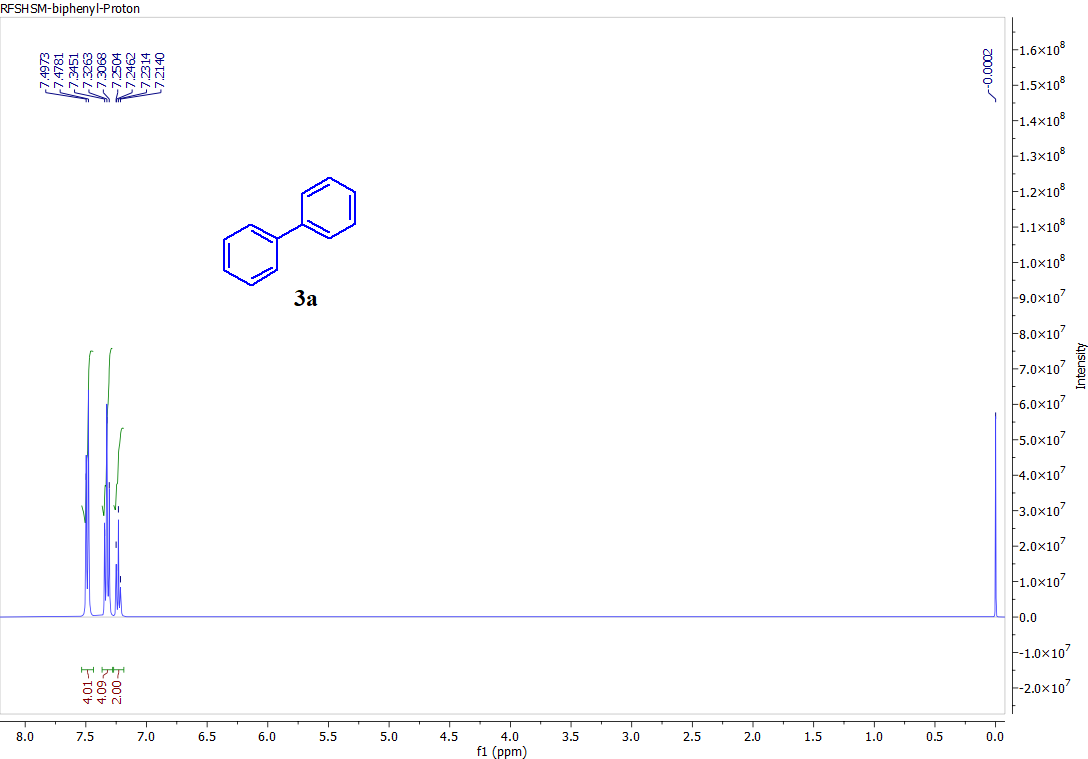


Fig. S9. ^1^H NMR spectra of Biphenyl (3a)


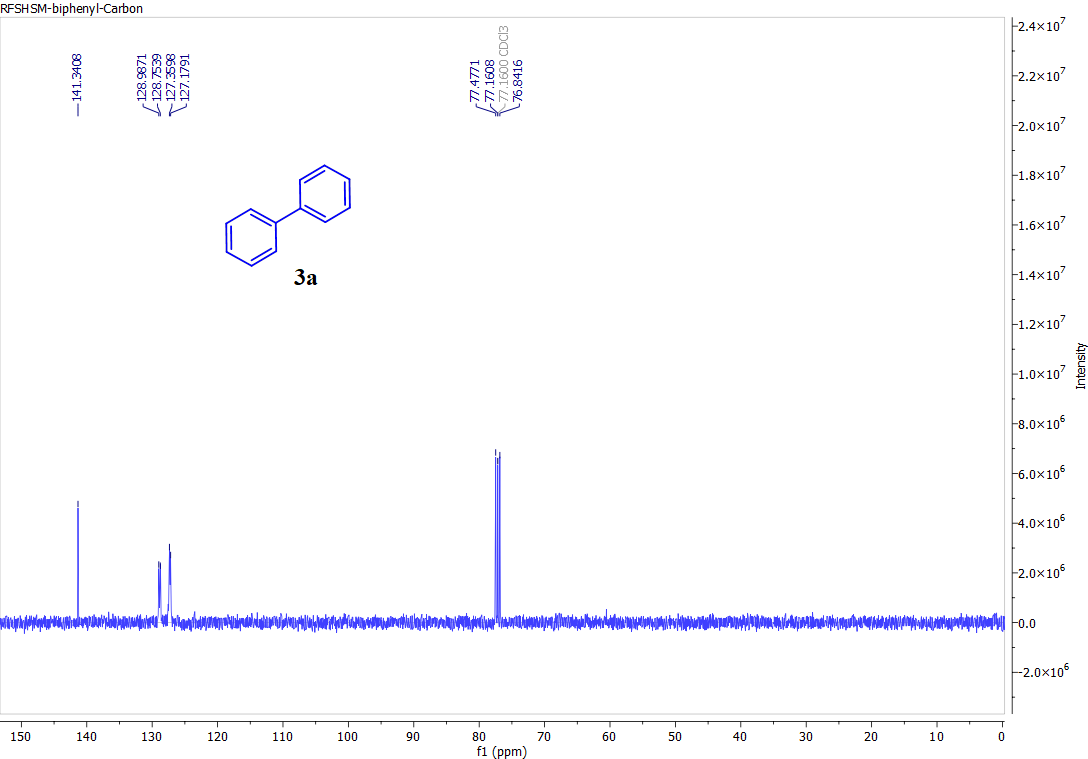


Fig. S10. ^13^C NMR spectra of Biphenyl (3a)


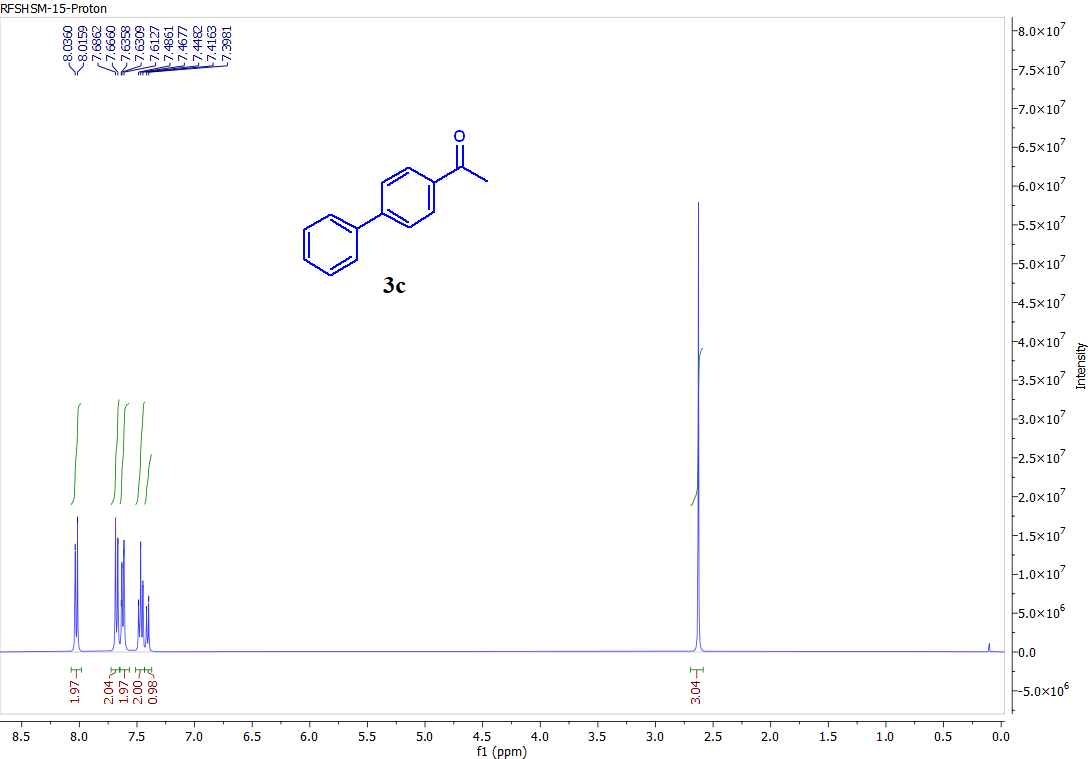


Fig. S11. ^1^H NMR spectra of 1-([1,1'-biphenyl]-4-yl)ethanone (**3c**)


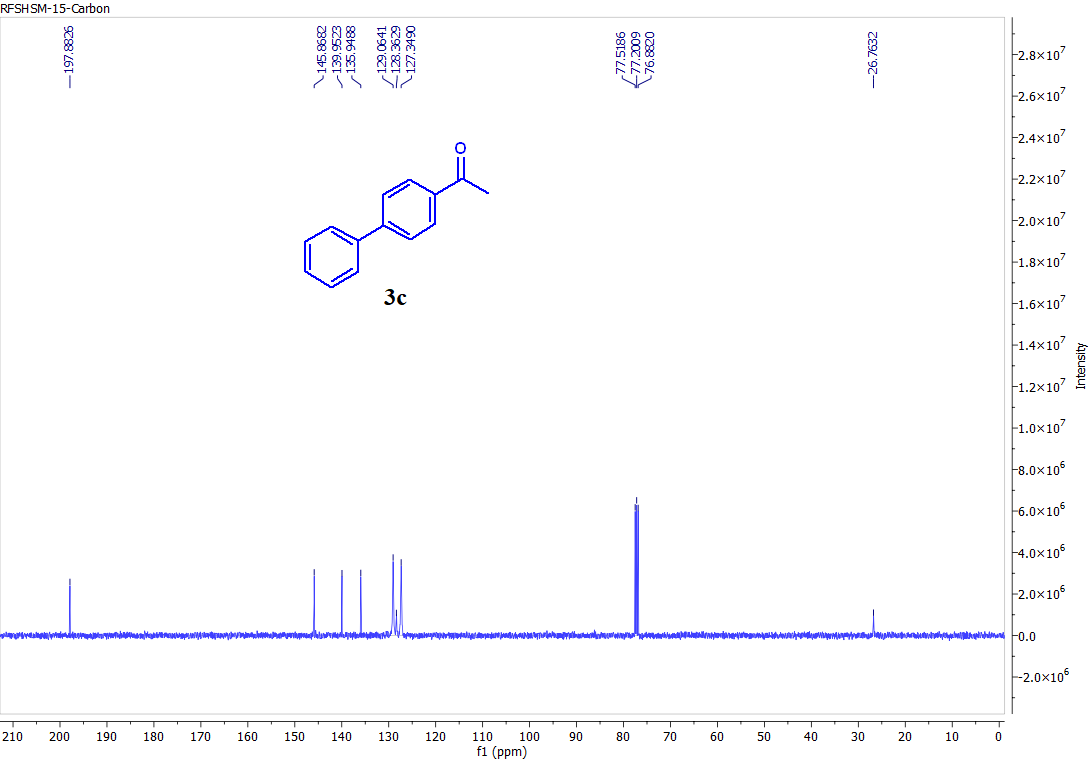


Fig. S12. ^13^C NMR spectra of 1-([1,1'-biphenyl]-4-yl)ethanone (**3c**)


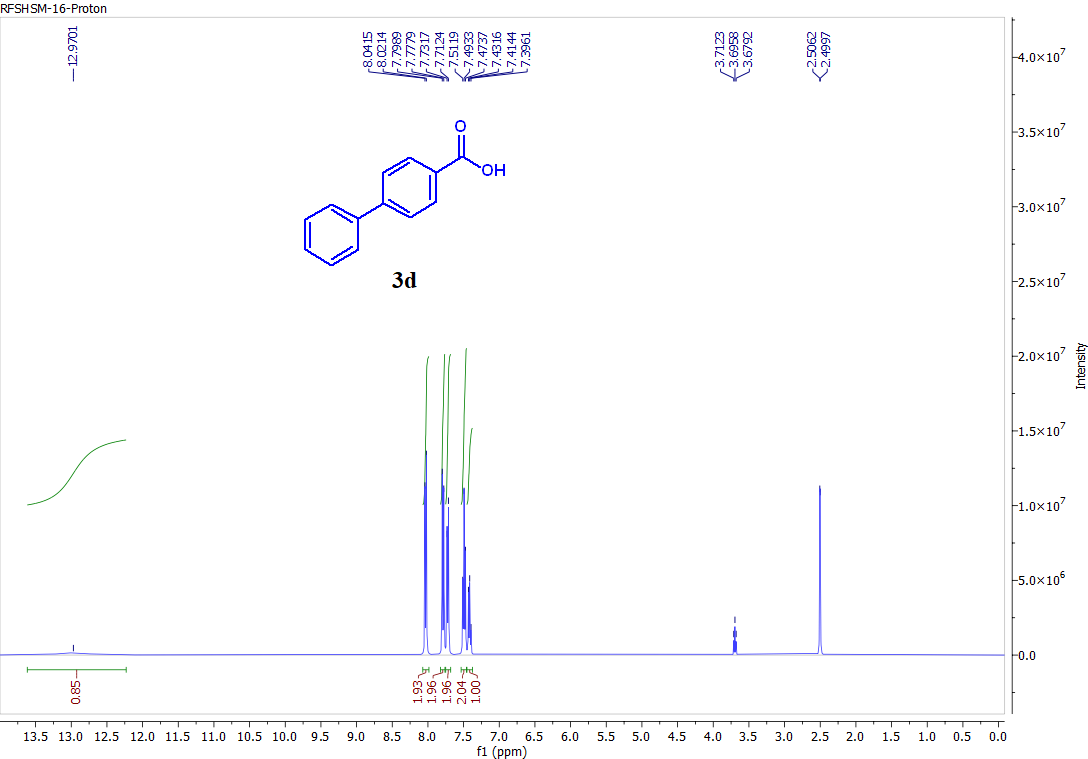


Fig. S13. ^1^H NMR spectra of [1,1'-biphenyl]-4-carboxylic acid (**3d**).


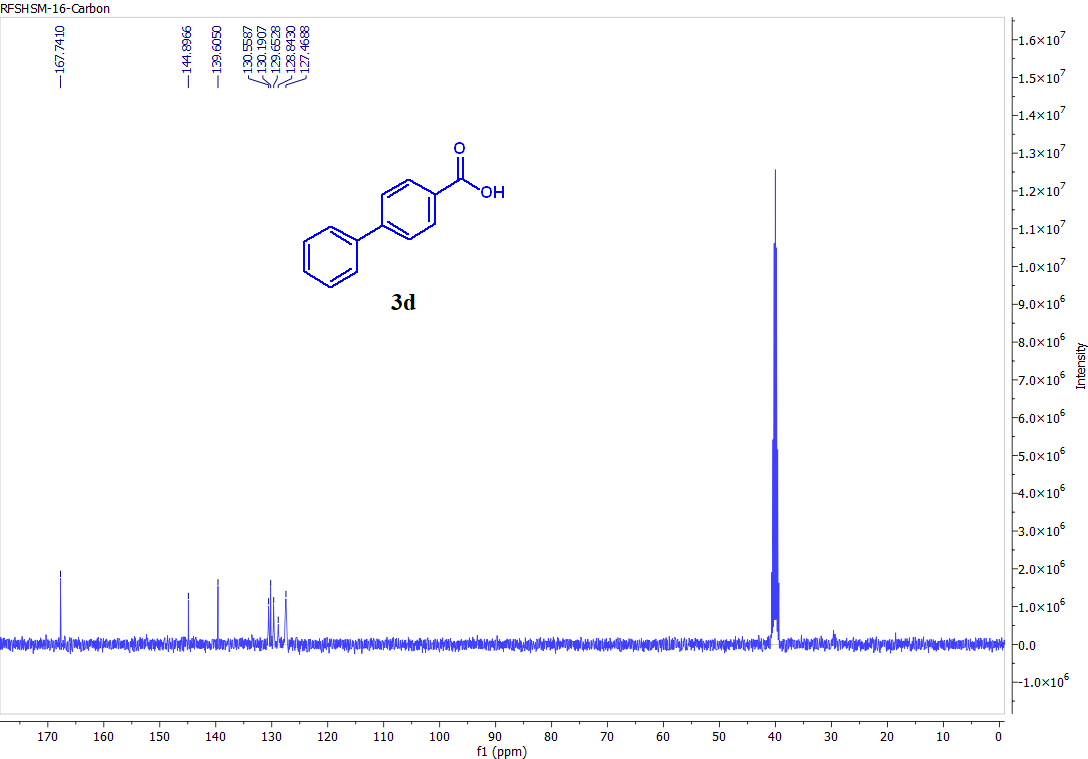


Fig. S14. ^13^C NMR spectra of [1,1'-biphenyl]-4-carboxylic acid (**3d**).


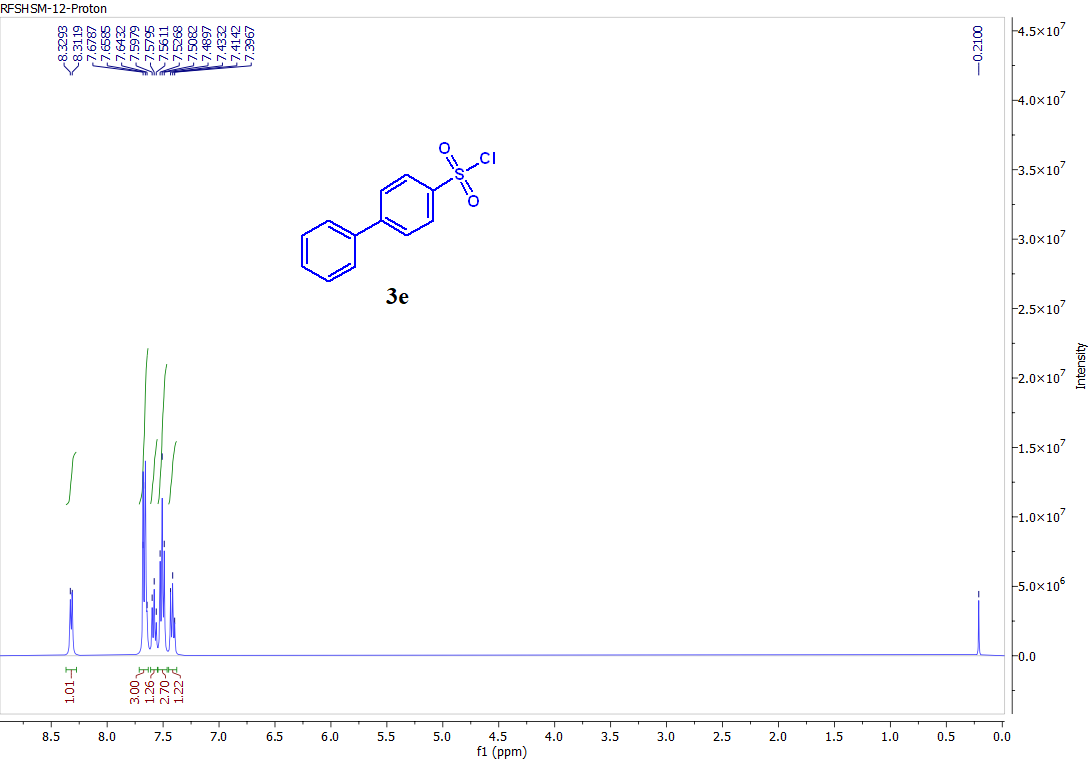


Fig. S15. ^1^H NMR spectra of [1,1'-biphenyl]-4-sulfonyl chloride (**3e**).


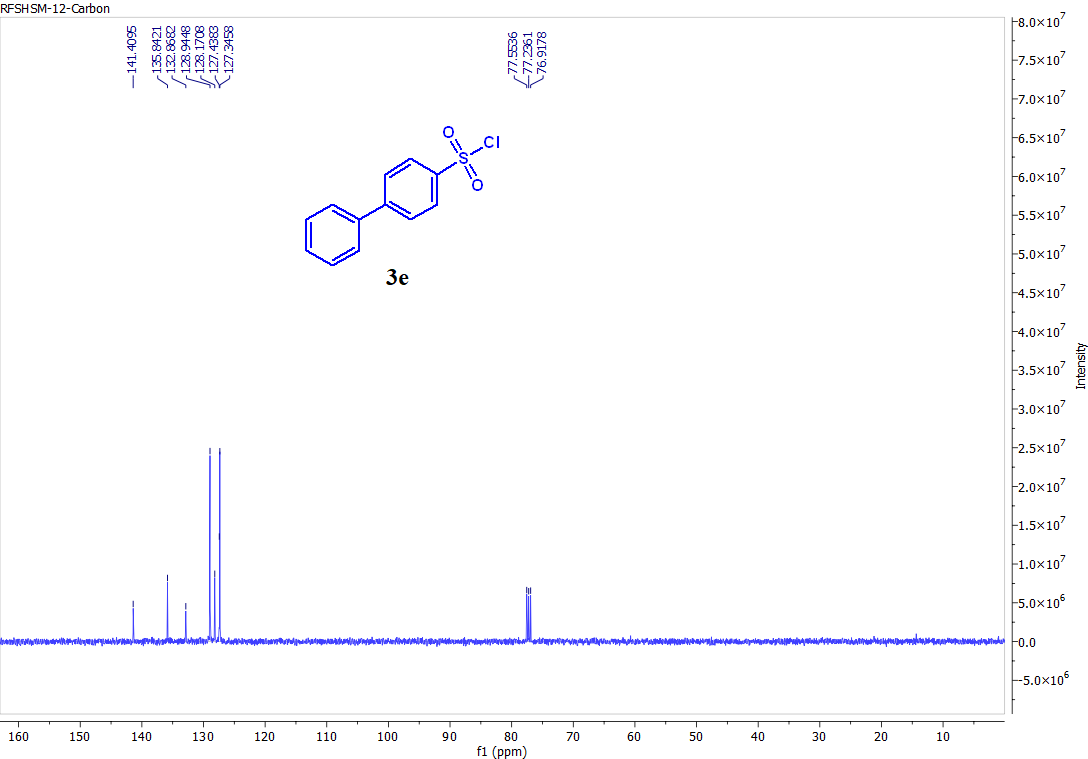


Fig. S16. ^13^C NMR spectra of [1,1'-biphenyl]-4-sulfonyl chloride (**3e**).


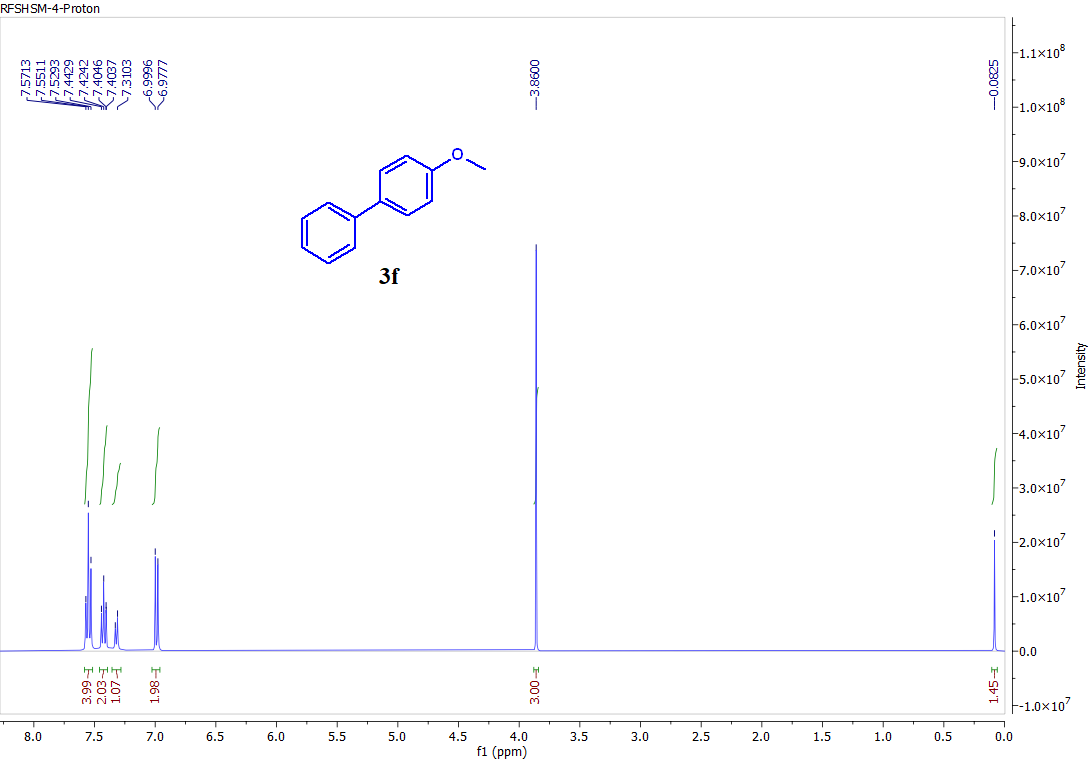


Fig. S17. ^1^H NMR spectra of 4-methoxy-1,1'-biphenyl (**3f**).


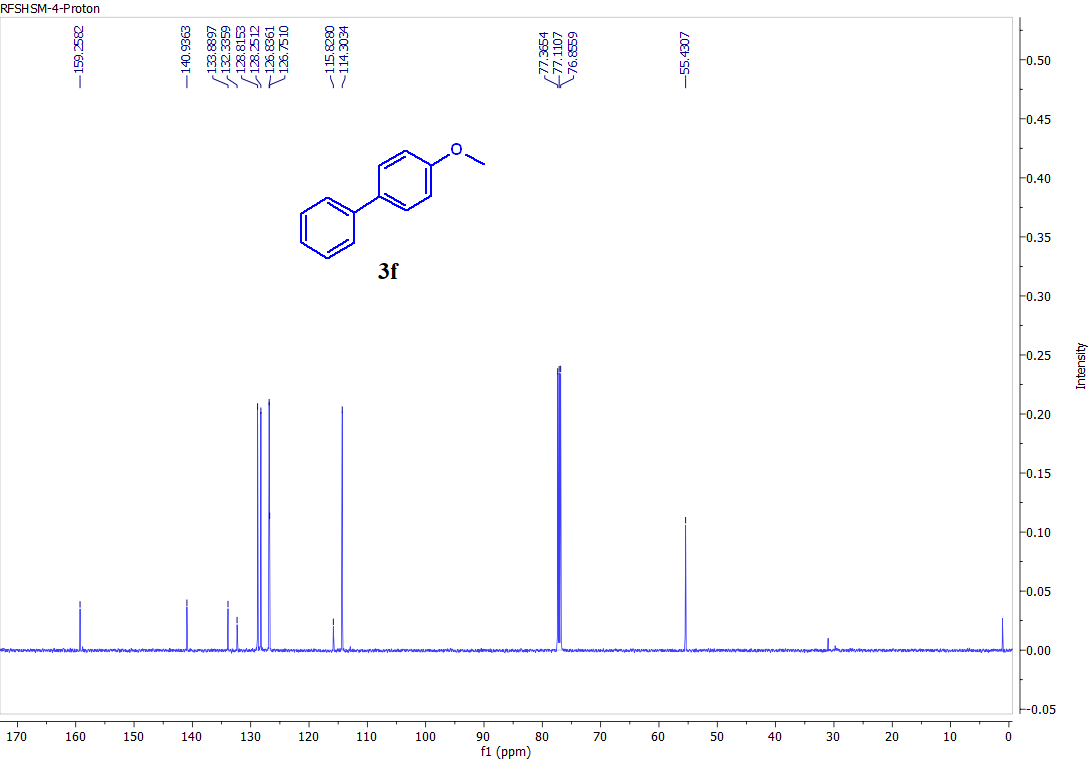


Fig. S18. ^13^C NMR spectra of 4-methoxy-1,1'-biphenyl (**3f**).


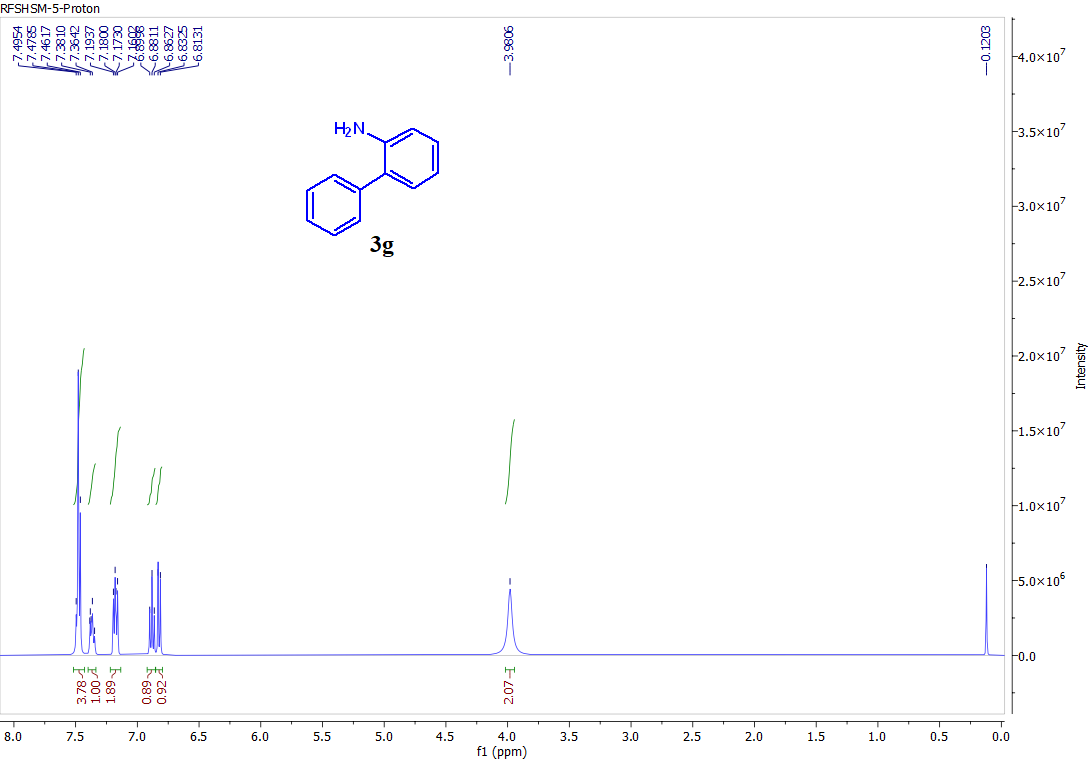


Fig. S19. ^1^H NMR spectra of [1,1'-biphenyl]-2-amine (**3g**).


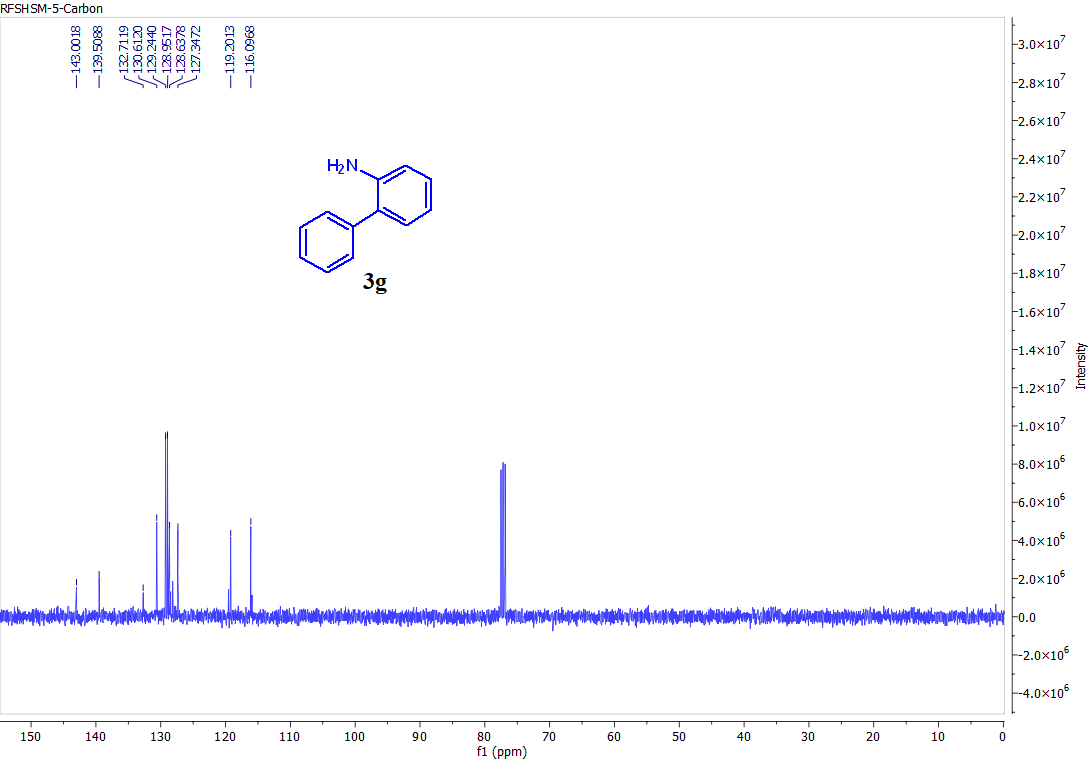


Fig. S20. ^13^C NMR spectra of [1,1'-biphenyl]-2-amine (**3g**).


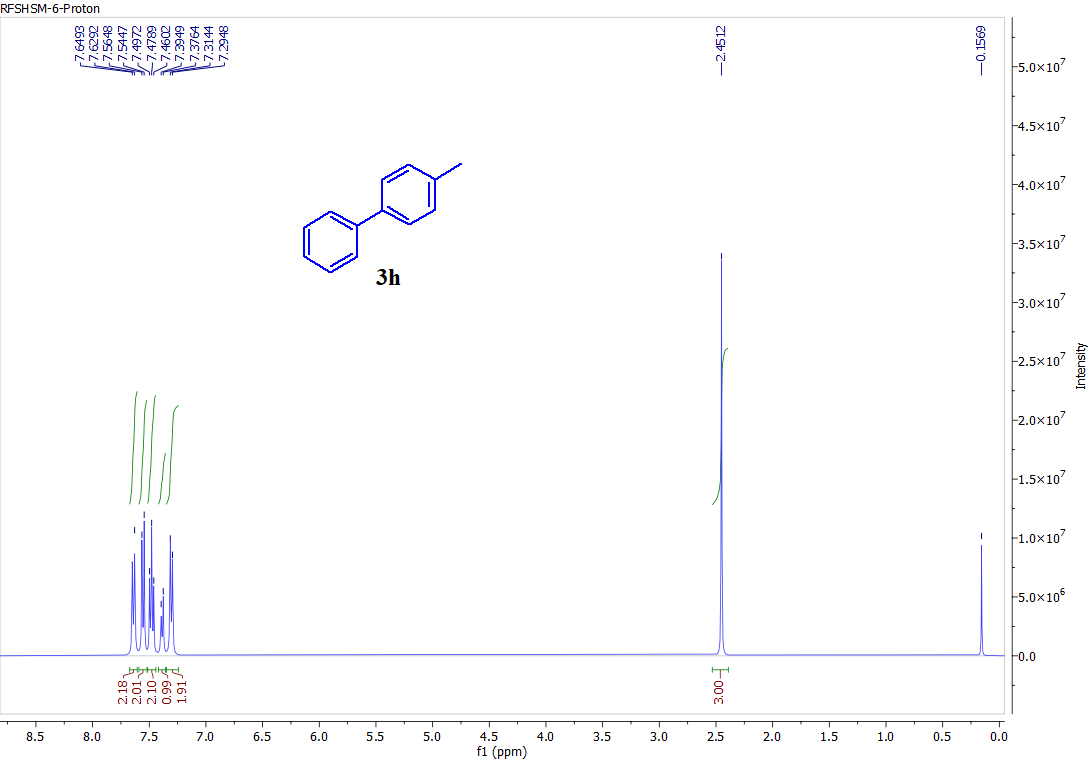


Fig. S21. ^1^H NMR spectra of 4-methyl-1,1'-biphenyl (**3h**).


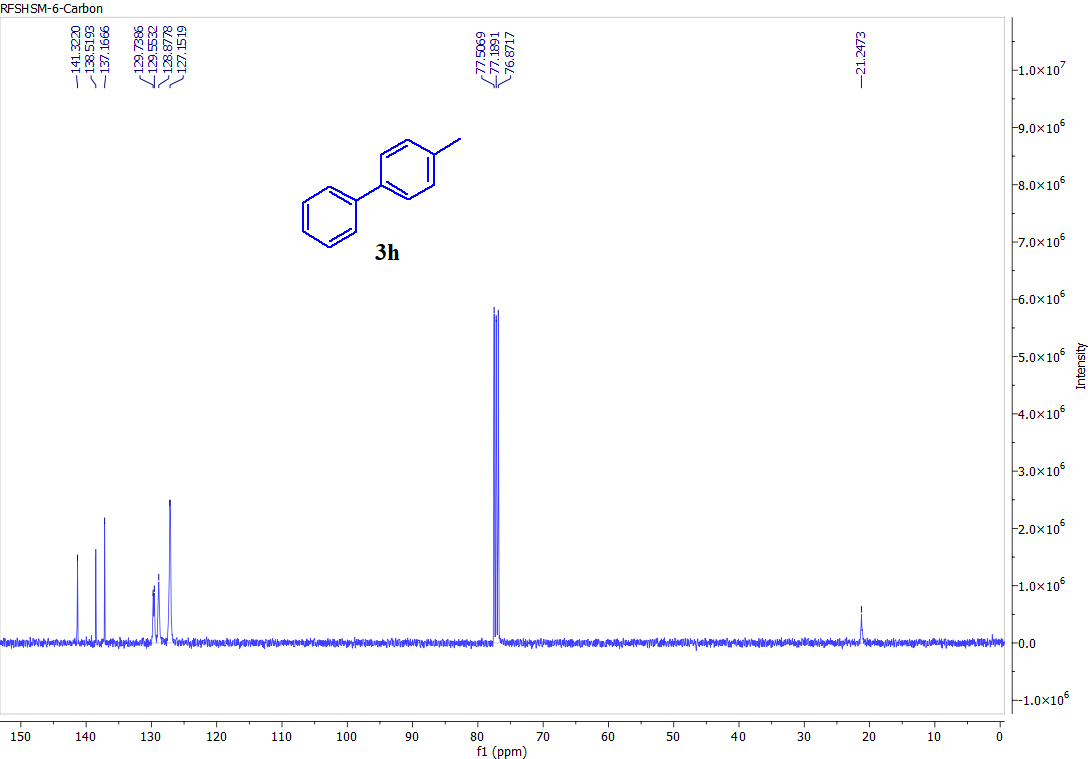


Fig. S22. ^13^C NMR spectra of 4-methyl-1,1'-biphenyl (**3h**).


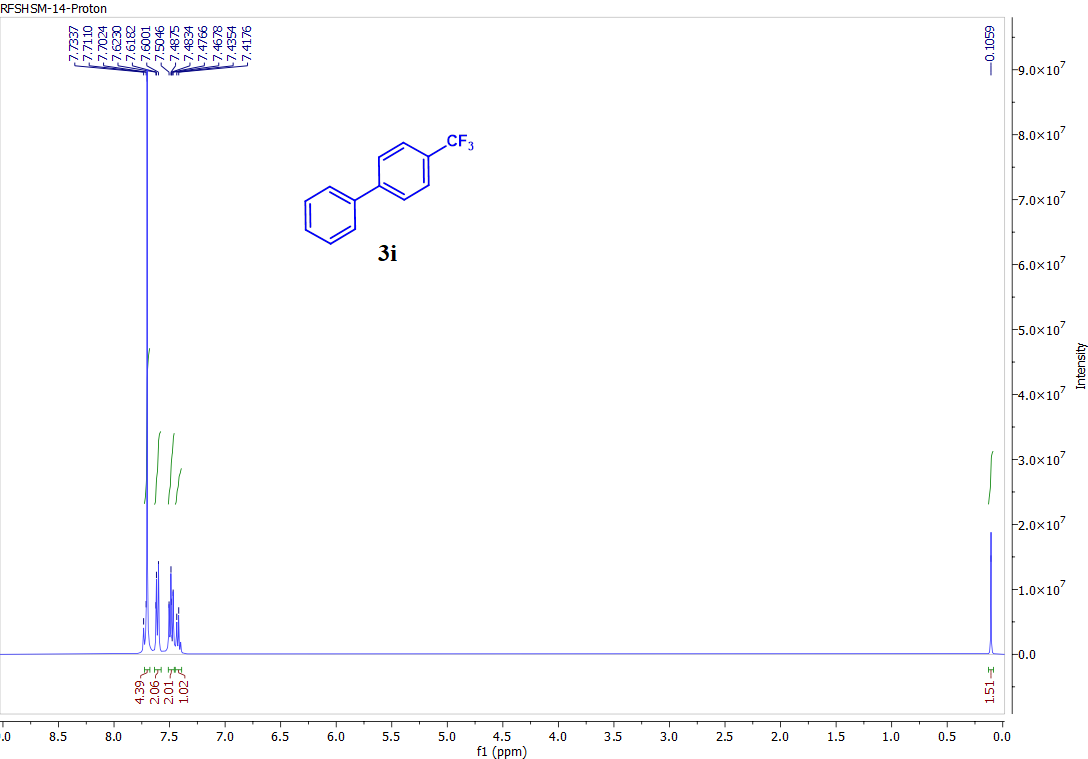


Fig. S23. ^1^H NMR spectra of 4-(trifluoromethyl)-1,1'-biphenyl (**3i**).


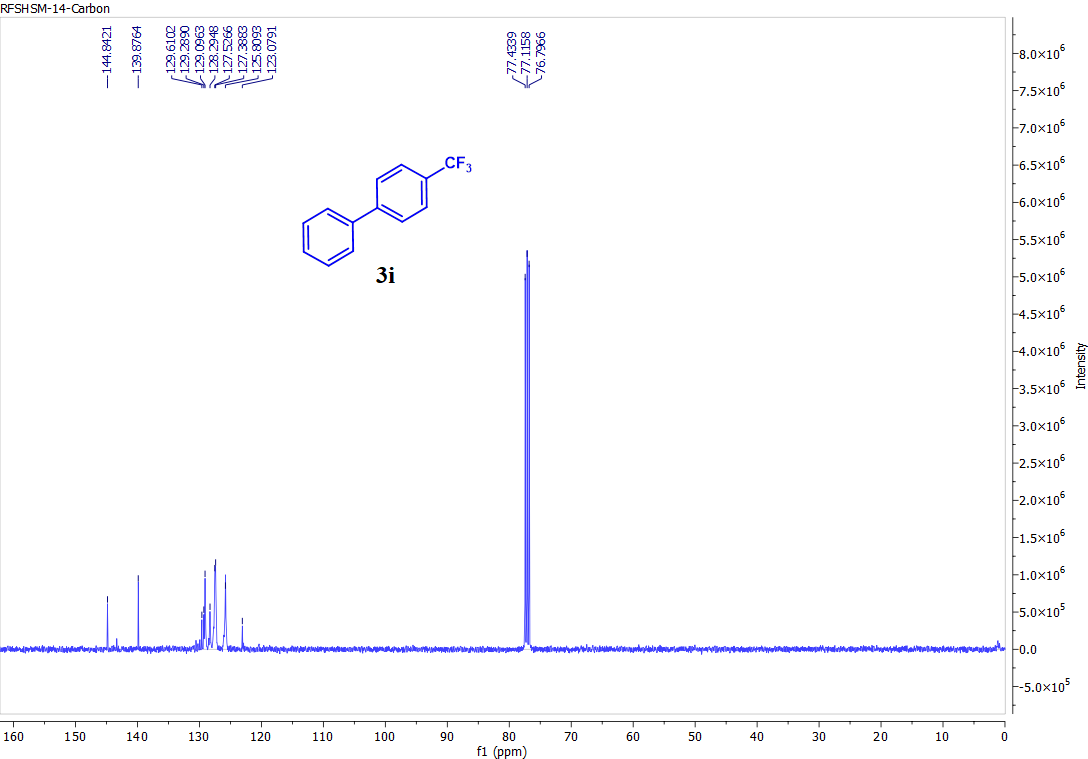


Fig. S24. ^13^C NMR spectra of 4-(trifluoromethyl)-1,1'-biphenyl (**3i**).


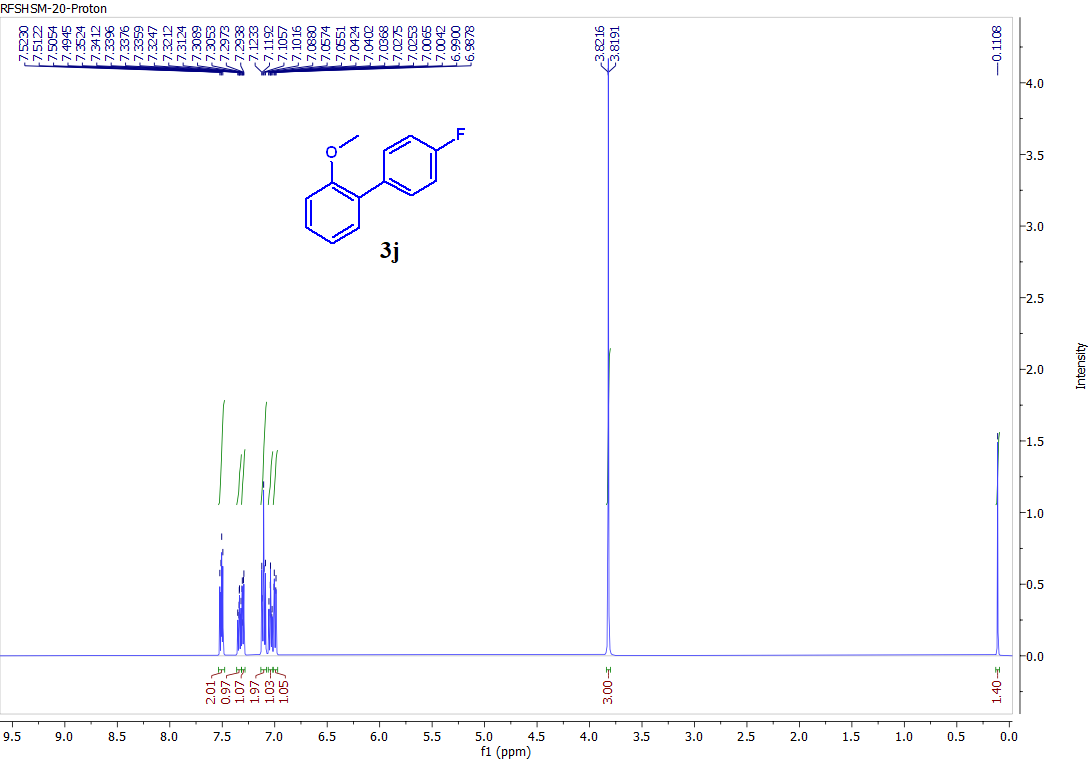


Fig. S25. ^1^H NMR spectra of 4'-fluoro-2-methoxy-1,1'-biphenyl (**3j**).


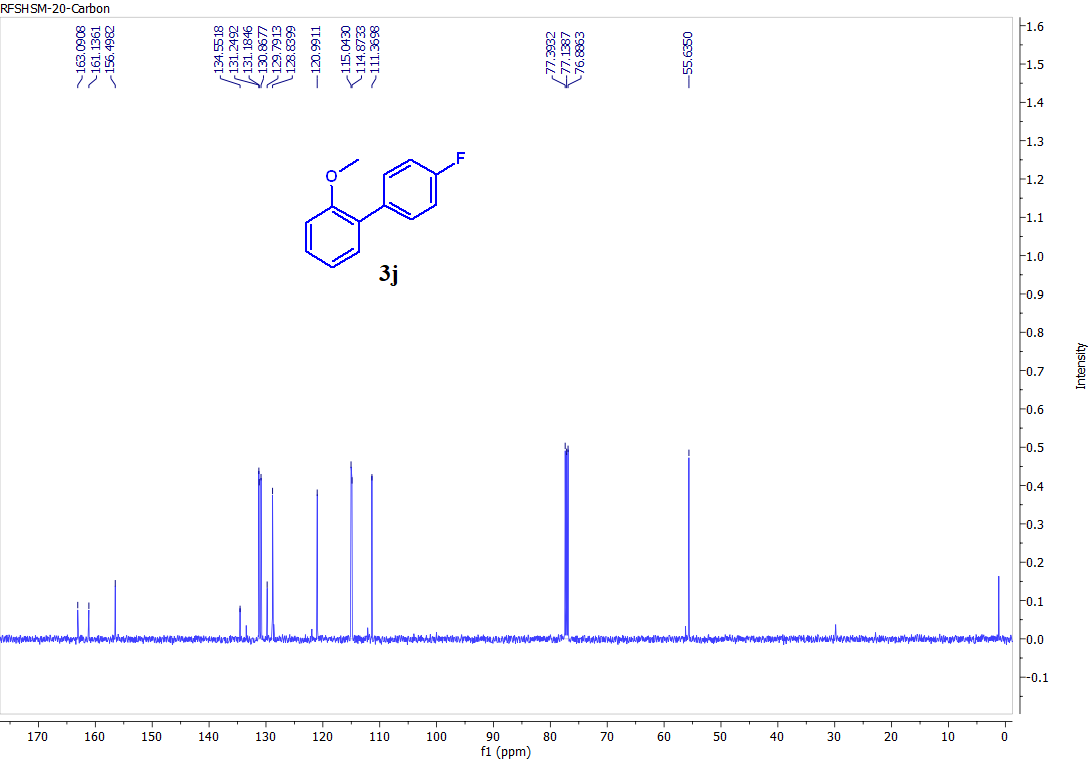


Fig. S26. ^13^C NMR spectra of 4'-fluoro-2-methoxy-1,1'-biphenyl (**3j**).


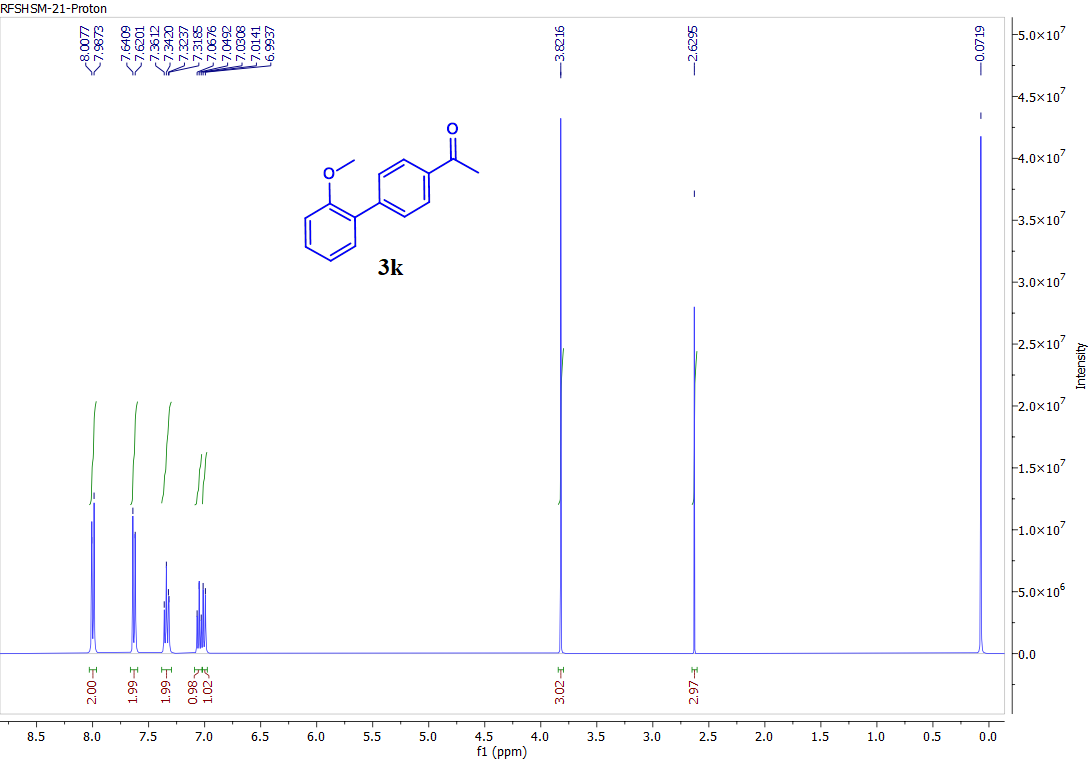


Fig. S27. ^1^H NMR spectra of 1-(2'-methoxy-[1,1'-biphenyl]-4-yl)ethanone (**3k**).


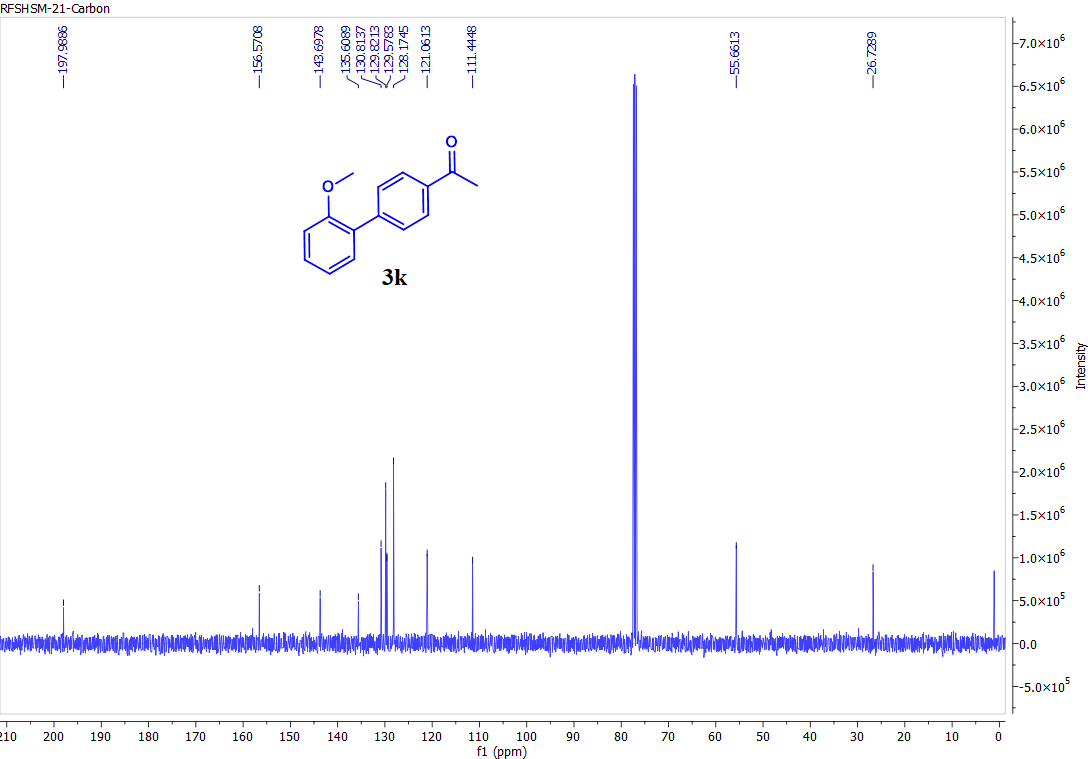


Fig. S28. ^13^C NMR spectra of 1-(2'-methoxy-[1,1'-biphenyl]-4-yl)ethanone (**3k**).


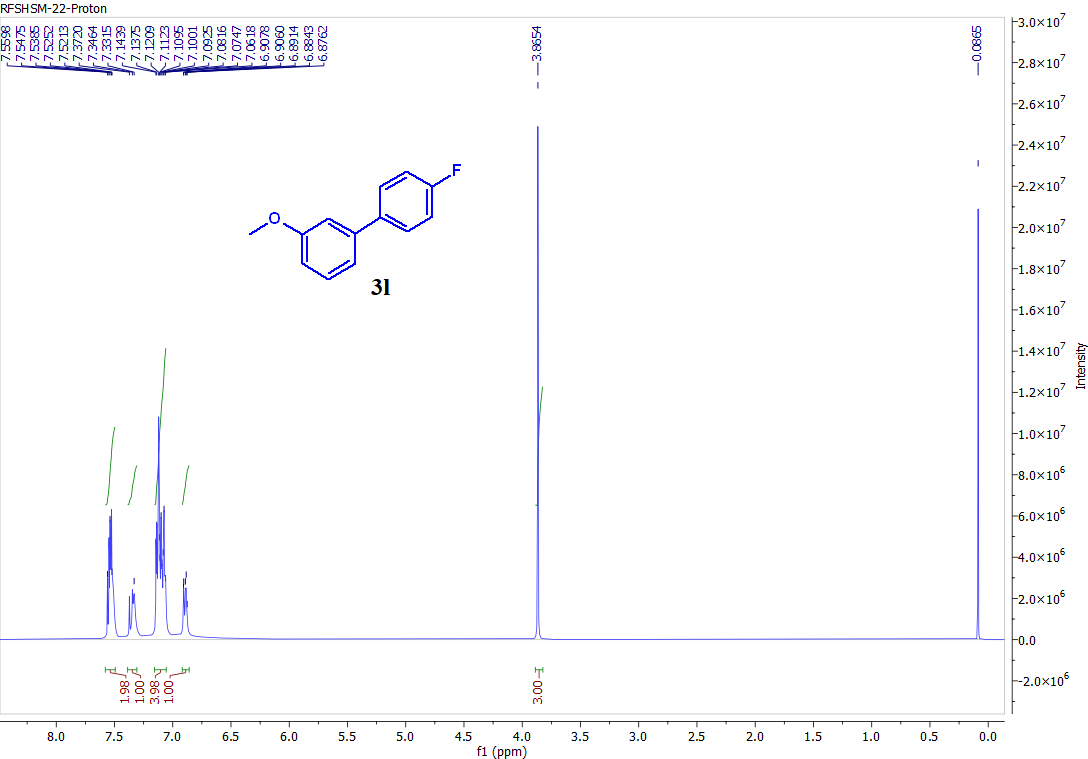


Fig. S29. ^1^H NMR spectra of 4'-fluoro-3-methoxy-1,1'-biphenyl (**3l**)


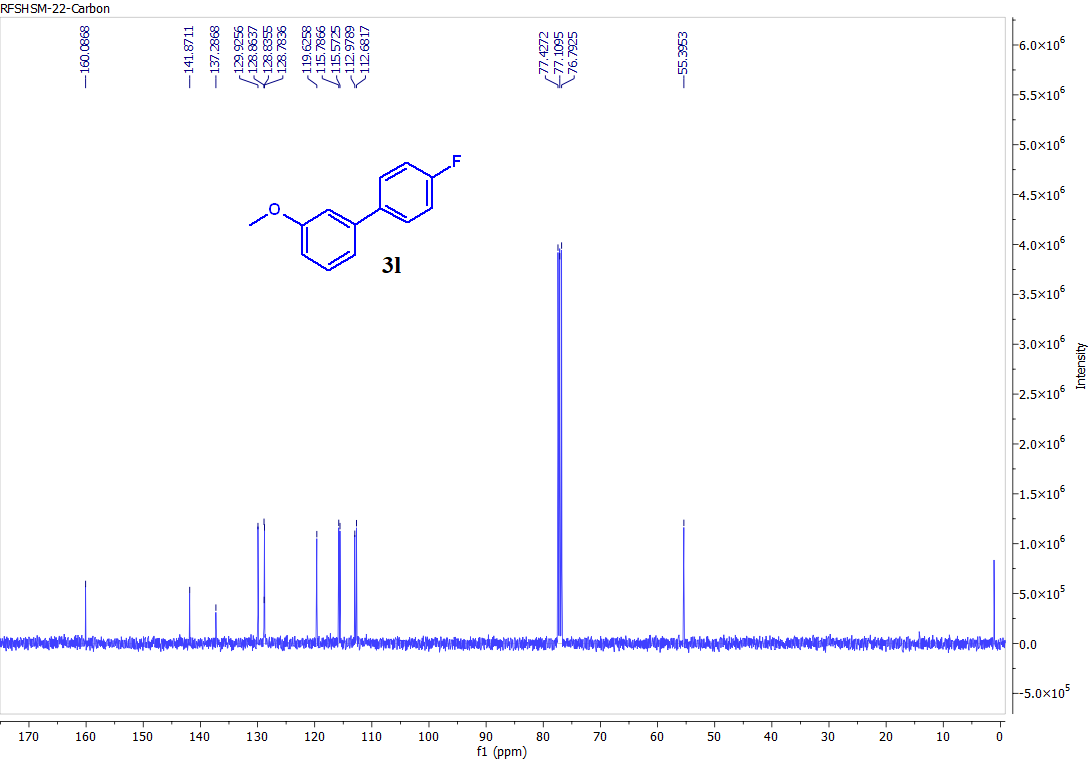


Fig. S30. ^13^C NMR spectra of 4'-fluoro-3-methoxy-1,1'-biphenyl (**3l**)


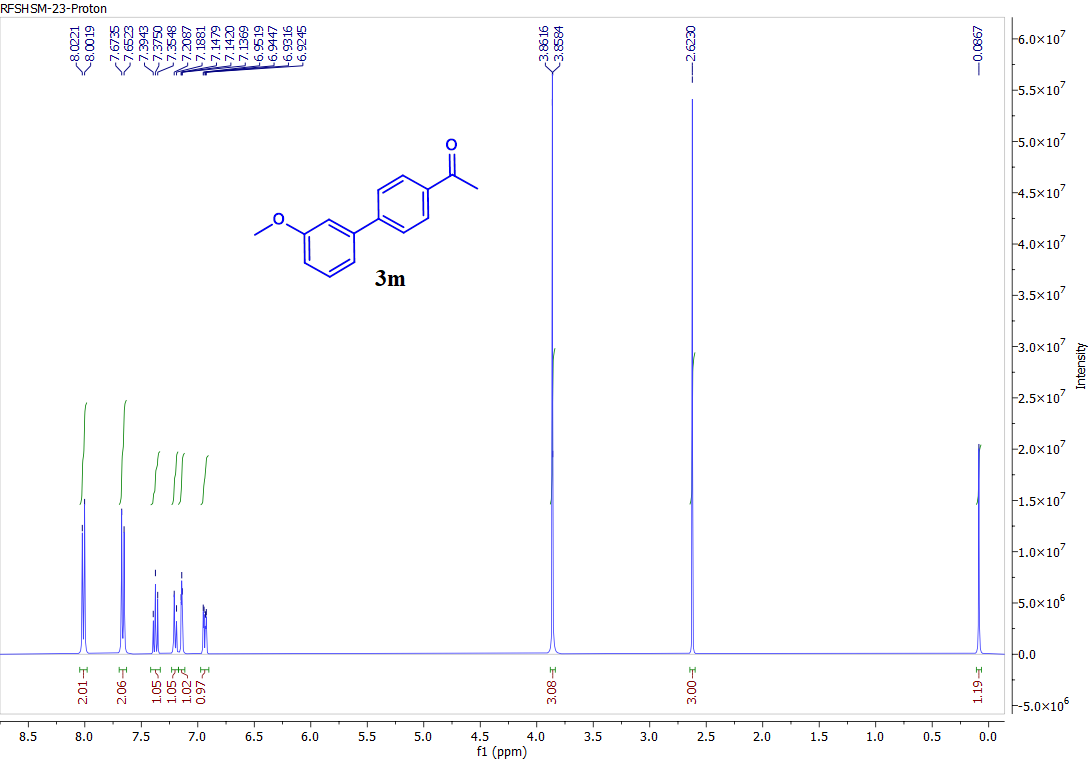


Fig. S31. ^1^H NMR spectra of 1-(3'-methoxy-[1,1'-biphenyl]-4-yl)ethanone (**3m**).


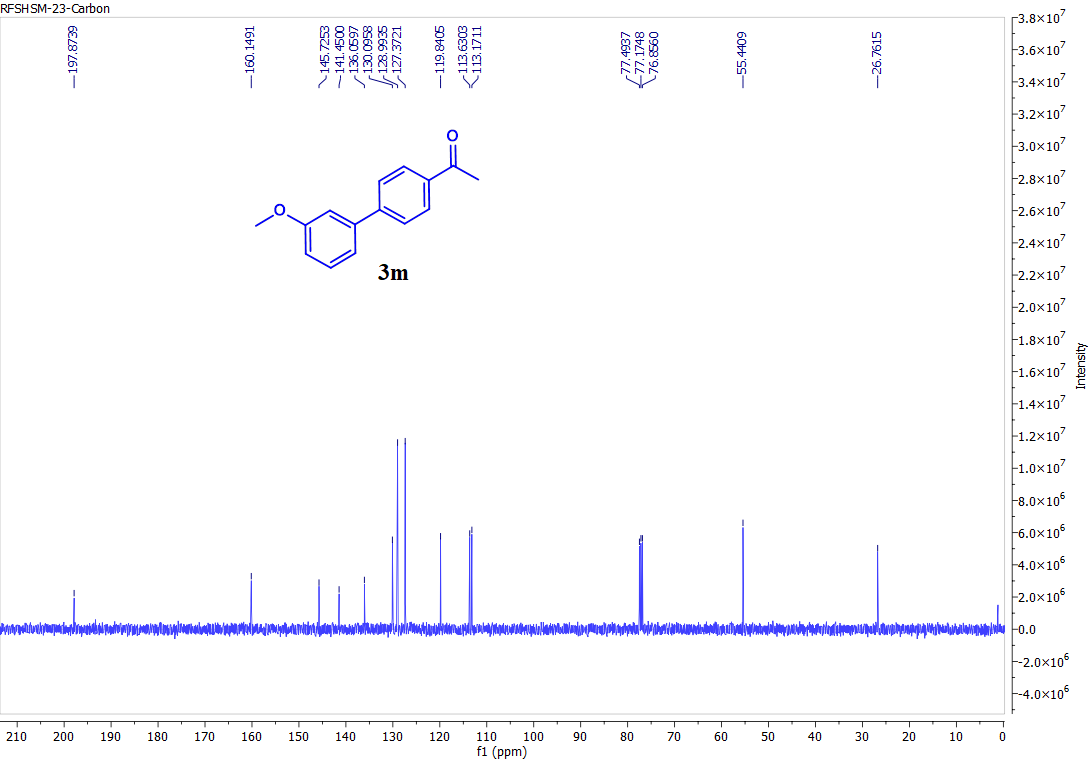


Fig. S32. ^13^C NMR spectra of 1-(3'-methoxy-[1,1'-biphenyl]-4-yl)ethanone (**3m**).


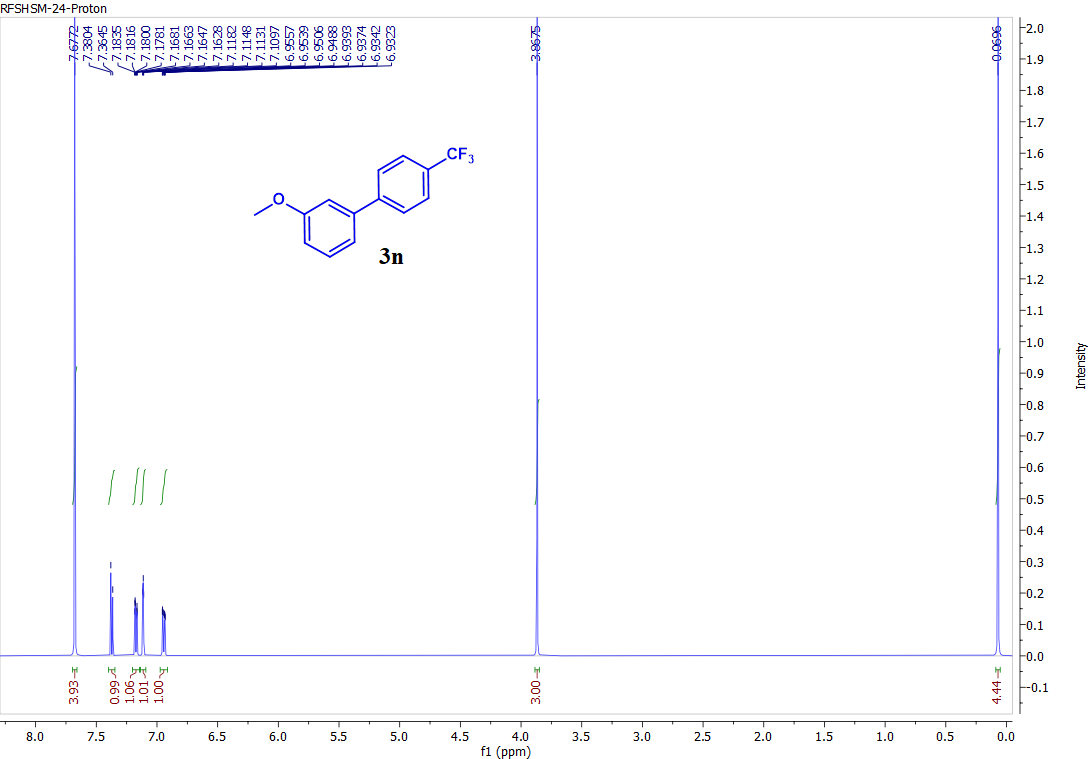


Fig. S33. ^1^H NMR spectra of 3-methoxy-4'-(trifluoromethyl)-1,1'-biphenyl (**3n**).


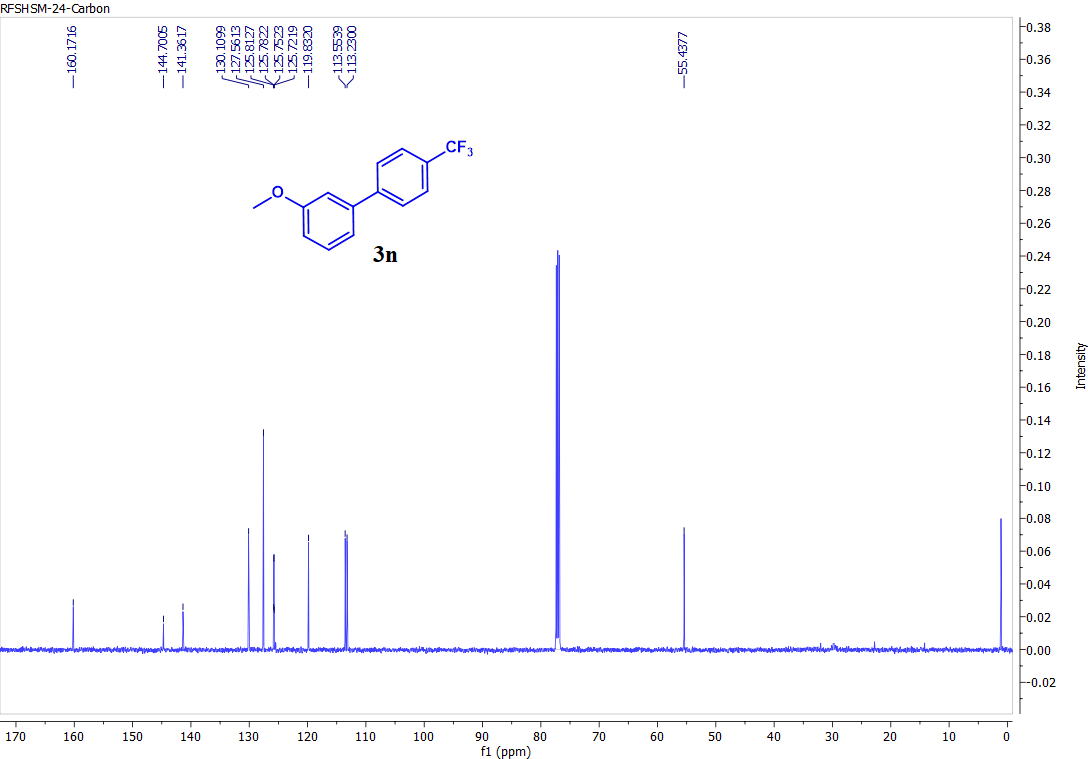


Fig. S34. ^13^C NMR spectra of 3-methoxy-4'-(trifluoromethyl)-1,1'-biphenyl (**3n**).


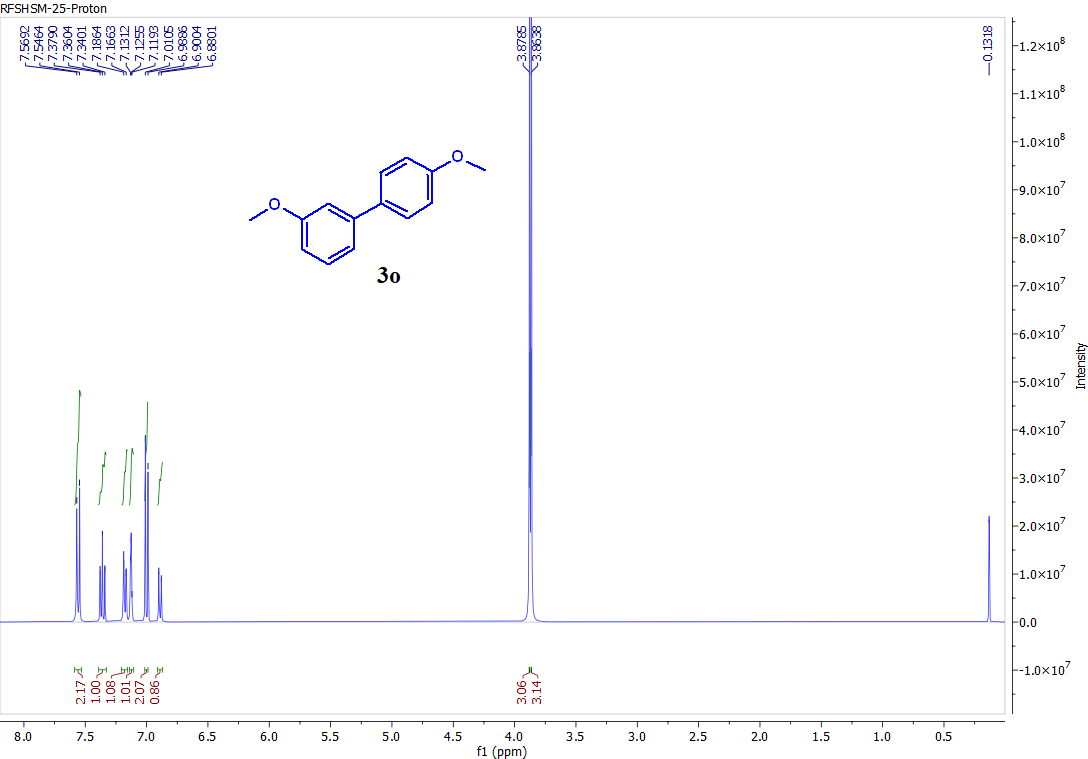


Fig. S35. ^1^H NMR spectra of 3,4'-dimethoxy-1,1'-biphenyl (**3o**).


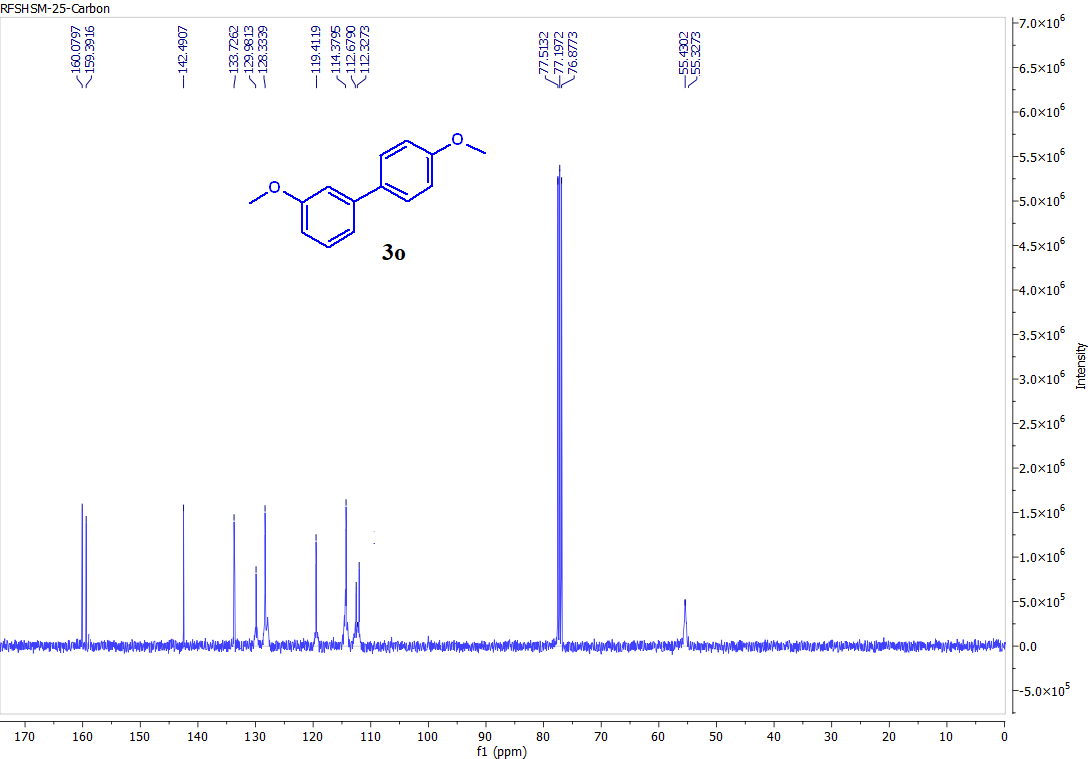


Fig. S36. ^13^C NMR spectra of 3,4'-dimethoxy-1,1'-biphenyl (**3o**).


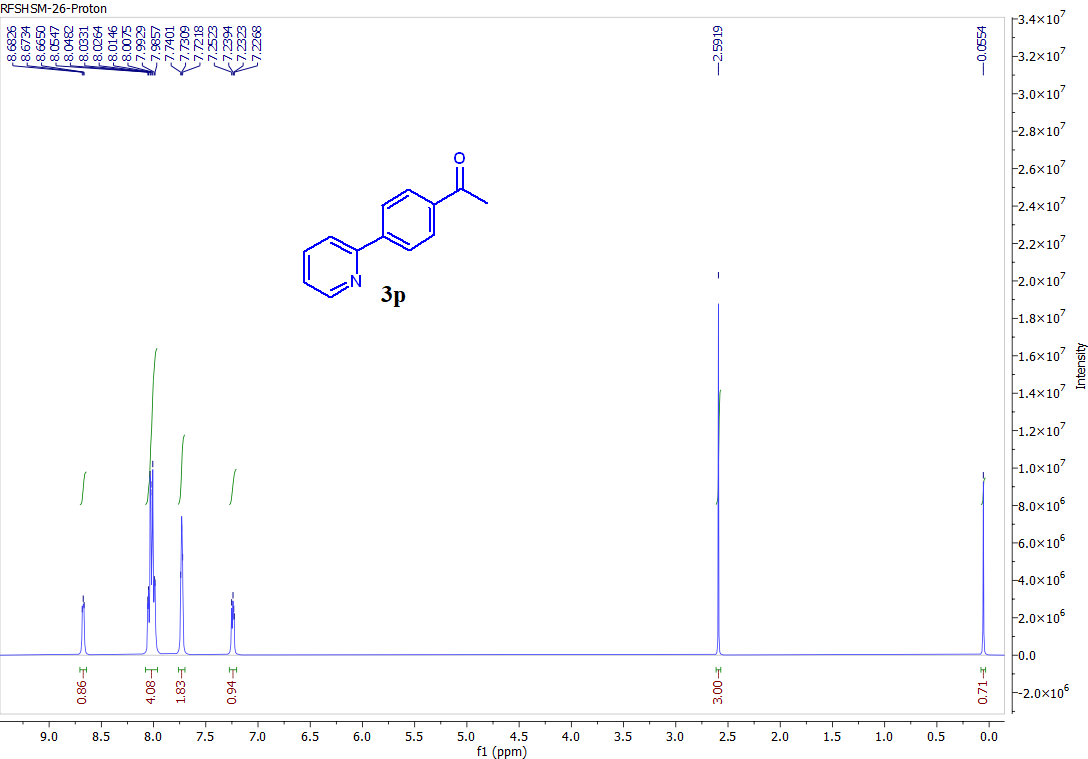


Fig. S37. ^1^H NMR spectra of 1-(4-(pyridin-2-yl)phenyl)ethanone (**3p**).


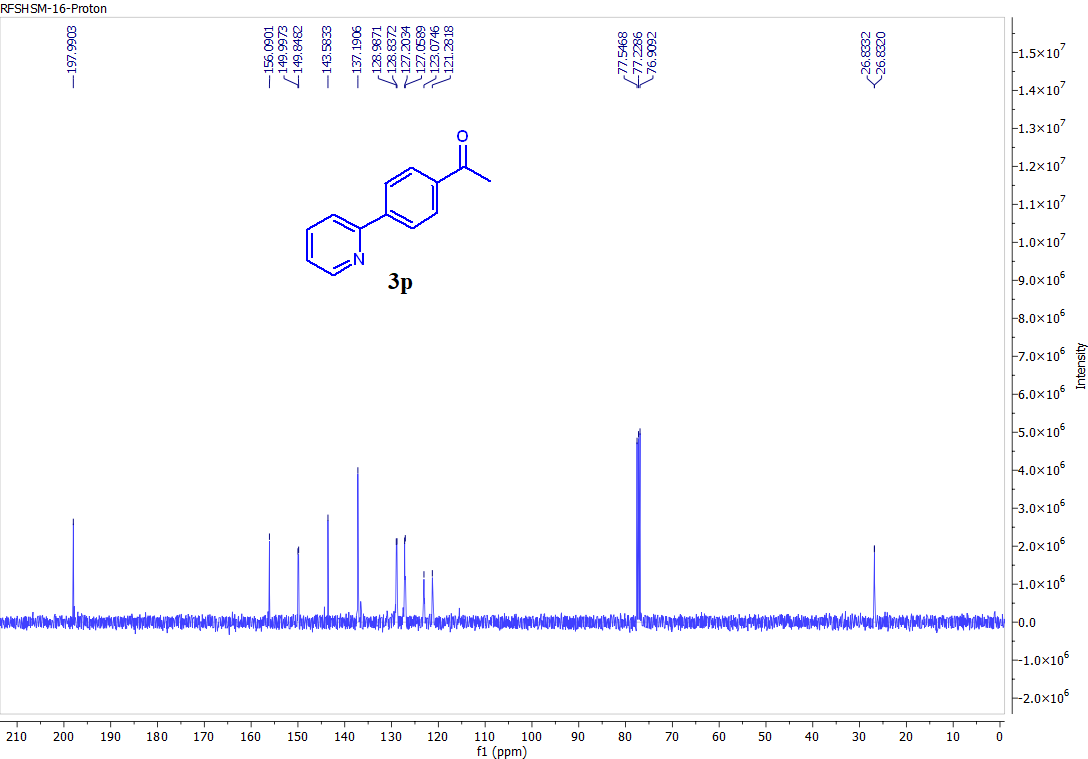


Fig. S38. ^13^C NMR spectra of 1-(4-(pyridin-2-yl)phenyl)ethanone (**3p**).

1. **General Information**: All aryl halides, all boronic acids, sodium dodecyl sulfate (SDS) and Potassium phospahate (K_3_PO_4_) were used as received from Sigma Aldrich. All bases were used as received (in air) or dried under vacuum at 100 °C (under inert atmosphere). Nanocatalyst **HRG-Py-Pd** and **HRG-Pd** were freshly prepared as described in the article. Flash chromatography was performed on 100-200 mesh silica gel. ^1^H and ^13^C Nuclear Magnetic Resonance (NMR) spectra were recorded on JEOL-400 MHz spectrometers at ambient temperature in CDCl_3_ & DMSO-*d*_6_ which were purchased from Sigma Aldrich. Chemical shifts (ppm) are referenced to the residual solvent peak. Coupling constants, *J*, are given in hertz. Abbreviations used in the designation of the signals: s = singlet, d = doublet, dd = doublet of doublets, ddd = doublet of doublet of doublets, dt = doublet of triplets, t = triplet, td = triplet of doublets, m = multiplet. Melting points were performed at London Metropolitan University.
2. **Typical Procedure for Suzuki reaction:** In a typical experiment, a mixture of sodium dodecyl sulfate (144 mg, 0.5 mmol), tripotassium phosphate (K_3_PO_4_, 399 mg), boronic acids **2a-f** (1.2 mmol) and deionized water (20 mL) were charged into a 100 mL round bottom flask. Aryl halide **1a- n** (1.0 mmol) was added to this mixture under stirring, followed by addition of pre-prepared nanocatalyst (**HRG-Py-Pd**/**HRG-Pd**) (5 mol %, 5.32 mg). The mixture was stirred at 100 ºC in an oil bath for 5-180 min and then extracted with ethyl acetate (3 X 20 mL). The combined organic extract was dried over anhydrous sodium sulfate (Na_2_SO_4_), the crude products were purified by column chromatography using (2-15%) Ethyl acetate/*n*-hexane to afford pure Suzuki Products (**3a-p**). In case of biphenyl product, the resulting mixture was analyzed by gas chromatography (GC), and ^1^H and ^13^C NMR. Biphenyl (**3a**): ^1^H NMR (400 MHz, CHLOROFORM-*D*) δ 7.49 (d, *J* = 7.7 Hz, 4H), 7.33 (t, *J* = 7.7 Hz, 4H), 7.23 (t, *J* = 7.3 Hz, 2H); ^13^C NMR (101 MHz, CHLOROFORM-*D*) δ 141.34, 128.99, 128.75, 127.36, 127.18.
3. **NMR data for all the synthesized compounds 3a-p.**
4. 1,1'-biphenyl (**3a**)

M.p.: 68-70 °C (lit.^1^ 69-71°C); ^1^H NMR (400 MHz, CHLOROFORM-*D*) δ 7.49 (d, *J* = 7.7 Hz, 4H, Ar-H), 7.33 (t, *J* = 7.7 Hz, 4H, Ar-H), 7.23 (t, *J* = 7.3 Hz, 2H, Ar-H); ^13^C NMR (101 MHz, CHLOROFORM-*D*) δ 141.34, 128.99, 128.75, 127.36, 127.18; Analytical data obtained were in agreement with the reported values^2^.

1. 4-phenylbenzophenone (**3b**)

M.p.: 101-102 °C. (lit.^3^ 102-103°C); ^1^H-NMR (400 MHz, CHLOROFORM-*D*): δ = 7.94-7.88 (m, 2H, Ar-H), 7.88-7.83 (m, 2H, Ar-H), 7.75-7.69 (m, 2H, Ar-H), 7.69-7.64 (m, 2H, Ar-H), 7.64-7.58 (m, 1H, Ar-H), 7.55-7.47 (m, 4H, Ar-H), 7.467.38 (m, 1H, Ar-H). ^13^C-NMR (101 MHz, CHLOROFORM-*D*): δ = 196.25, 145.16, 139.91, 137.71, 136.18, 132.30, 130.66, 129.93, 128.91, 128.24, 128.13, 127.23, 126.90; Spectral data match those reported in the literature^4^.

1. 1-([1,1'-biphenyl]-4-yl)ethanone (**3c**)

M.p.: 119-1121 °C. (lit.^5^ 121-123°C); ^1^H NMR (400 MHz, CHLOROFORM-*D*) δ 8.03 (d, *J* = 8.0 Hz, 2H, Ar-H), 7.68 (d, *J* = 8.1 Hz, 2H, Ar-H), 7.62 (d, *J* = 7.3 Hz, 2H, Ar-H), 7.47 (t, *J* = 7.6 Hz, 2H, Ar-H), 7.41 (d, *J* = 7.3 Hz, 1H, Ar-H), 2.63 (s, 3H, COCH_3_); ^13^C NMR (101 MHz, CHLOROFORM-*D*) δ 197.88, 145.87, 139.95, 135.95, 129.06, 128.36, 127.35, 26.76; Spectral data match those reported in the literature^6^.

1. [1,1'-biphenyl]-4-carboxylic acid (**3d**)

M.p.: 224-225 °C. (lit.^7^ 226-227°C); ^1^H NMR (400 MHz, DMSO-*D*_6_) δ 12.97 (s, 1H, COOH), 8.03 (d, *J* = 8.0 Hz, 2H, Ar-H), 7.79 (d, *J* = 8.4 Hz, 2H, Ar-H), 7.72 (d, *J* = 7.7 Hz, 2H, Ar-H), 7.49 (t, *J* = 7.6 Hz, 2H, Ar-H), 7.41 (t, *J* = 7.1 Hz, 1H, Ar-H); ^13^C NMR (101 MHz, DMSO-*D*_6_) δ 167.74, 144.90, 139.61, 130.56, 130.19, 129.65, 128.84, 127.47; Spectral data match those reported in the literature^8^.

1. [1,1'-biphenyl]-4-sulfonyl chloride (**3e**)

M.p.: 114-116 °C. (lit.^9^ 114-115°C); ^1^H NMR (400 MHz, CHLOROFORM-*D*) δ 8.32 (d, *J* = 7.0 Hz, 1H, Ar-H), 7.67 (d, *J* = 8.1 Hz, 3H, Ar-H), 7.58 (t, *J* = 7.3 Hz, 1H, Ar-H), 7.51 (t, *J* = 7.4 Hz, 3H, Ar-H), 7.41 (t, *J* = 7.3 Hz, 1H, Ar-H). ^13^C NMR (101 MHz, CHLOROFORM-*D*) δ 141.41, 135.84, 132.87, 128.94, 128.17, 127.44, 127.35; Spectral data match those reported in the literature^10^.

1. 4-methoxy-1,1'-biphenyl (**3f**)

M.p.: 89-90 °C. (Lit.^11^ 88-90 °C); ^1^H NMR (400 MHz, CHLOROFORM-*D*) δ 7.55 (t, *J* = 8.4 Hz, 4H, Ar-H), 7.42 (t, *J* = 7.7 Hz, 2H, Ar-H), 7.32 (d, *J* = 7.4 Hz, 1H, Ar-H), 6.99 (d, *J* = 8.8 Hz, 2H, Ar-H), 3.86 (s, 3H, OCH_3_); ^13^C NMR (126 MHz, CHLOROFORM-*D*) δ 159.26, 140.94, 133.89, 132.34, 128.82, 128.25, 126.84, 126.75, 115.83, 114.30, 55.43. Analytical data obtained were in agreement with the reported values^12^.

1. [1,1'-biphenyl]-2-amine (**3g**)

M.p.: 49-51 °C. (Lit.^13^ 53-54 °C); ^1^H NMR (400 MHz, CHLOROFORM-*D*) δ 7.47 (d, *J* = 6.8 Hz, 4H, Ar-H), 7.40 – 7.34 (m, 1H, Ar-H), 7.18 (dd, *J* = 8.1, 5.3 Hz, 2H, Ar-H), 6.88 (t, *J* = 7.4 Hz, 1H, Ar-H), 6.82 (d, *J* = 7.7 Hz, 1H, Ar-H), 3.98 (s, 2H, NH_2_); ^13^C NMR (101 MHz, CHLOROFORM-*D*) δ 143.00, 139.51, 132.71, 130.61, 129.24, 128.95, 128.64, 127.35, 119.20, 116.10. Analytical data obtained were in agreement with the reported values^14^.

1. 4-methyl-1,1'-biphenyl (**3h**)

M.p.: 48-50 °C. (Lit.^15^ 47-48 °C); ^1^H NMR (400 MHz, CHLOROFORM-*D*) δ 7.64 (d, *J* = 8.0 Hz, 2H, Ar-H), 7.55 (d, *J* = 8.0 Hz, 2H, Ar-H), 7.48 (t, *J* = 7.4 Hz, 2H, Ar-H), 7.39 (d, *J* = 7.4 Hz, 1H, Ar-H), 7.30 (d, *J* = 7.8 Hz, 2H, Ar-H), 2.45 (s, 3H, CH_3_). ^13^C NMR (101 MHz, CHLOROFORM-*D*) δ 141.32, 138.52, 137.17, 129.74, 129.55, 128.88, 127.15, 21.25; Spectral data match those reported in the literature^2^.

1. 4-(trifluoromethyl)-1,1'-biphenyl (**3i**)

M.p.: 69-71 °C. (Lit.^16^ 70 °C); ^1^H NMR (400 MHz, CHLOROFORM-*D*) δ 7.70 (s, 4H, Ar-H), 7.64 – 7.58 (m, 2H, Ar-H), 7.49 (dd, *J* = 8.2, 6.5 Hz, 2H, Ar-H), 7.42 (t, *J* = 7.2 Hz, 1H, Ar-H). ^13^C NMR (101 MHz, CHLOROFORM-*D*) δ 144.84, 139.88, 129.61, 129.29, 129.10, 128.29, 127.53, 127.39, 125.81, 123.08; Spectral data match those reported in the literature^2,17^.

1. 10. 4'-fluoro-2-methoxy-1,1'-biphenyl (**3j**)

M.p.: 198-201 °C; ^1^H NMR (500 MHz, CHLOROFORM-*D*) δ 7.54 – 7.48 (m, 2H, Ar-H), 7.36 – 7.32 (m, 1H, Ar-H), 7.30 (dd, *J* = 7.5, 1.8 Hz, 1H, Ar-H), 7.13 – 7.08 (m, 2H, Ar-H), 7.04 (ddd, *J* = 7.5, 6.6, 1.1 Hz, 1H, Ar-H), 7.00 (dt, *J* = 8.1, 1.3 Hz, 1H, Ar-H), 3.82 (d, *J* = 1.2 Hz, 3H, OCH); ^13^C NMR (126 MHz, CHLOROFORM-*D*) δ 163.09, 161.14, 156.50, 134.55, 131.25, 131.18, 130.87, 129.79, 128.84, 120.99, 115.04, 114.87, 111.37, 55.63. Spectral data match those reported in the literature^18^.

1. 1-(2'-methoxy-[1,1'-biphenyl]-4-yl)ethanone (**3k**)

M.p.: 106-108 °C. (Lit.^19^ 108-109 °C);^1^H NMR (400 MHz, CHLOROFORM-*D*) δ 8.00 (d, *J* = 8.1 Hz, 2H, Ar-H), 7.63 (d, *J* = 8.3 Hz, 2H, Ar-H), 7.38 – 7.30 (m, 2H, Ar-H), 7.05 (t, *J* = 7.4 Hz, 1H, Ar-H), 7.00 (d, *J* = 8.2 Hz, 1H, Ar-H), 3.82 (s, 3H, OCH_3_), 2.63 (s, 3H, CH_3_); ^13^C NMR (101 MHz, CHLOROFORM-*D*) δ 197.99, 156.57, 143.70, 135.61, 130.81, 129.82, 129.58, 128.17, 121.06, 111.44, 55.66, 26.73. Spectral data match those reported in the literature^19^.

1. 4'-fluoro-3-methoxy-1,1'-biphenyl (**3l**)

M.p.: 203-204 °C; ^1^H NMR (400 MHz, CHLOROFORM-*D*) δ 7.54 (dt, *J* = 6.9, 3.3 Hz, 2H, Ar-H), 7.39 – 7.31 (m, 1H, Ar-H), 7.16 – 7.06 (m, 4H, Ar-H), 6.92 – 6.86 (m, 1H, Ar-H), 3.87 (s, 3H, OCH_3_); ^13^C NMR (101 MHz, CHLOROFORM-*D*) δ 160.09, 141.87, 137.29, 129.93, 128.86, 128.84, 128.78, 119.63, 115.79, 115.57, 112.98, 112.68, 55.40. Spectral data match those reported in the literature^20^.

1. 1-(3'-methoxy-[1,1'-biphenyl]-4-yl)ethanone (**3m**)

M.p.: 50-52 °C. (Lit.^21^ 51-52 °C); ^1^H NMR (400 MHz, CHLOROFORM-*D*) δ 8.01 (d, *J* = 8.1 Hz, 2H, Ar-H), 7.66 (d, *J* = 8.5 Hz, 2H, Ar-H), 7.42 – 7.33 (m, 1H, Ar-H), 7.20 (d, *J* = 8.2 Hz, 1H, Ar-H), 7.14 (t, *J* = 2.2 Hz, 1H, Ar-H), 6.94 (dd, *J* = 8.1, 2.8 Hz, 1H, Ar-H), 3.86 (s, 3H, OCH_3_), 2.62 (s, 3H, CH_3_); ^13^C NMR (101 MHz, CHLOROFORM-*D*) δ 197.87, 160.15, 145.73, 141.45, 136.06, 130.10, 128.99, 127.37, 119.84, 113.63, 113.17, 55.44, 26.76. Spectral data match those reported in the literature^22^.

1. 3-methoxy-4'-(trifluoromethyl)-1,1'-biphenyl (**3n**)

M.p.: 156-158 °C. (Lit.^23^ 156-157 °C);^1^H NMR (500 MHz, CHLOROFORM-*D*) δ 7.68 (s, 4H, Ar-H), 7.37 (d, *J* = 7.9 Hz, 1H, Ar-H), 7.17 (ddd, *J* = 7.7, 1.8, 1.0 Hz, 1H, Ar-H), 7.11 (dd, *J* = 2.6, 1.7 Hz, 1H, Ar-H), 6.94 (ddd, *J* = 8.2, 2.6, 0.9 Hz, 1H, Ar-H), 3.87 (s, 3H, OCH_3_); ^13^C NMR (126 MHz, CHLOROFORM-*D*) δ 160.17, 144.70, 141.36, 130.11, 127.56, 125.81, 125.78, 125.75, 125.72, 119.83, 113.55, 113.23, 55.44. Analytical data obtained were in agreement with the reported values^24^.

1. 3,4'-dimethoxy-1,1'-biphenyl (**3o**)

M.p.: 58-60 °C. (Lit.^25^ 60-61 °C); ^1^H NMR (400 MHz, CHLOROFORM-*D*) δ 7.56 (d, *J* = 9.1 Hz, 2H, Ar-H), 7.36 (t, *J* = 7.8 Hz, 1H, Ar-H), 7.18 (d, *J* = 8.0 Hz, 1H, Ar-H), 7.13 (d, *J* = 2.3 Hz, 1H, Ar-H), 7.00 (d, *J* = 8.7 Hz, 2H, Ar-H), 6.89 (d, *J* = 8.1 Hz, 1H, Ar-H), 3.88 (s, 3H, OCH_3_), 3.86 (s, 3H, OCH_3_); ^13^C NMR (101 MHz, CHLOROFORM-*D*) δ 160.08, 159.39, 142.49, 133.73, 129.98, 128.33, 119.41, 114.38, 112.68, 112.33, 77.51, 77.20, 76.88, 55.43, 55.32; Analytical data obtained were in agreement with the reported values^26^.

1. 1-(4-(pyridin-2-yl)phenyl)ethanone (**3p**)

M.p.: 194-195 °C;^1^H NMR (400 MHz, CHLOROFORM-*D*) δ 8.67 (t, *J* = 3.5 Hz, 1H, Ar-H), 8.02 (qd, *J* = 8.7, 2.7 Hz, 4H, Ar-H), 7.73 (d, *J* = 3.6 Hz, 2H, Ar-H), 7.27 – 7.20 (m, 1H, Ar-H), 2.59 (s, 3H, COCH_3_); ^13^C NMR (101 MHz, CHLOROFORM-*D*) δ 197.99, 156.09, 150.00, 149.85, 143.58, 137.19, 128.99, 128.84, 127.20, 127.06, 123.07, 121.28, 26.83, 26.83. Analytical data obtained were in agreement with the reported values^12^.

1 Tamura, Y., Chun, M.-W., Inoue, K. & Minamikawa, J. A novel synthesis of biaryls from diaryliodonium salts. *Synthesis* **1978**, 822-822 (1978).

2 Bandari, R., Höche, T., Prager, A., Dirnberger, K. & Buchmeiser, M. R. Ring‐Opening Metathesis Polymerization Based Pore‐Size‐Selective Functionalization of Glycidyl Methacrylate Based Monolithic Media: Access to Size‐Stable Nanoparticles for Ligand‐Free Metal Catalysis. *Chem. Eur. J.* **16**, 4650-4658 (2010).

3 Keumi, T., Yoshimura, K., Shimada, M. & Kitajima, H. 2-(Trifluoromethylsulfonyloxy) pyridine as a reagent for the ketone synthesis from carboxylic acids and aromatic hydrocarbons. *Bull. Chem. Soc. Jpn.* **61**, 455-459 (1988).

4 Sapountzis, I., Lin, W., Kofink, C. C., Despotopoulou, C. & Knochel, P. Iron‐Catalyzed Aryl–Aryl Cross‐Couplings with Magnesium‐Derived Copper Reagents. *Angew. Chem. Int. Ed.* **44**, 1654-1658 (2005).

5 Sharghi, H., Jokar, M., Doroodmand, M. M. & Khalifeh, R. Catalytic Friedel–Crafts acylation and benzoylation of aromatic compounds using activated hematite as a novel heterogeneous catalyst. *Adv. Synth. Catal.* **352**, 3031-3044 (2010).

6 Fairlamb, I. J., Kapdi, A. R. & Lee, A. F. η2-dba Complexes of Pd (0): The substituent effect in Suzuki− Miyaura coupling. *Org. Lett.* **6**, 4435-4438 (2004).

7 Berger, P., Bessmernykh, A., Caille, J.-C. & Mignonac, S. Palladium-catalyzed hydroxycarbonylation of aryl and vinyl bromides by mixed acetic formic anhydride. *Synthesis* **2006**, 3106-3110 (2006).

8 Du, Z., Zhou, W., Wang, F. & Wang, J.-X. In situ generation of palladium nanoparticles: ligand-free palladium catalyzed ultrafast Suzuki–Miyaura cross-coupling reaction in aqueous phase at room temperature. *Tetrahedron* **67**, 4914-4918 (2011).

9 Oneto, J. F. & Way, E. Sulfophenylarsonic Acids and Certain of their Derivatives. V. p-(p-Sulfophenyl)-and p-(p-Sulfonamidophenyl)-phenylarsonic Acids1. *J. Am. Chem. Soc.* **63**, 3068-3070 (1941).

10 Jautze, S., Seiler, P. & Peters, R. Synthesis of Nearly Enantiopure Allylic Amines by Aza‐Claisen Rearrangement of Z‐Configured Allylic Trifluoroacetimidates Catalyzed by Highly Active Ferrocenylbispalladacycles. *Chem. Eur. J.* **14**, 1430-1444 (2008).

11 Badone, D., Baroni, M., Cardamone, R., Ielmini, A. & Guzzi, U. Highly efficient palladium-catalyzed boronic acid coupling reactions in water: scope and limitations. *J. Org. Chem.* **62**, 7170-7173 (1997).

12 Schneider, S. & Bannwarth, W. Application of the fluorous biphase concept to palladium‐catalyzed Suzuki couplings. *Helv. Chim. Acta* **84**, 735-742 (2001).

13 McLaughlin, M. A. & Barnes, D. M. A practical and selective reduction of nitroarenes using elemental sulfur and mild base. *Tetrahedron Lett.* **47**, 9095-9097 (2006).

14 Schmidt, A. & Rahimi, A. A versatile catalyst system for Suzuki–Miyaura syntheses of sterically hindered biaryls employing a cyclobutene-1, 2-bis (imidazolium) salt. *Chem. Commun.* **46**, 2995-2997 (2010).

15 Riggleman, S. & DeShong, P. Application of silicon-based cross-coupling technology to triflates. *J. Org. Chem.* **68**, 8106-8109 (2003).

16 Hey, D., Saunders, F. & Williams, G. H. 110. Homolytic aromatic substitution. Part XXI. The arylation of benzotrihalides. *J. Chem. Soc.*, 554-562 (1961).

17 Ackermann, L., Potukuchi, H. K., Althammer, A., Born, R. & Mayer, P. Tetra-ortho-Substituted Biaryls through Palladium-Catalyzed Suzuki− Miyaura Couplings with a Diaminochlorophosphine Ligand. *Org. Lett.* **12**, 1004-1007 (2010).

18 Beadle, J. R., Korzeniowski, S. H., Rosenberg, D. E., Garcia-Slanga, B. J. & Gokel, G. W. Phase-transfer-catalyzed Gomberg-Bachmann synthesis of unsymmetrical biarenes: a survey of catalysts and substrates. *J. Org. Chem.* **49**, 1594-1603 (1984).

19 Zhang, G. Ligand-free Suzuki–Miyaura reaction catalysed by Pd/C at room temperature. *J. Chem. Res.* **2004**, 593-595 (2004).

20 Ackermann, L. & Althammer, A. Air-stable PinP (O) H as preligand for palladium-catalyzed Kumada couplings of unactivated tosylates. *Org. Lett.* **8**, 3457-3460 (2006).

21 Bradsher, C. K., Brown, F. C. & Porter, H. K. Synthesis and Fungistatic Activity of Some 3-Hydroxybiphenyl Derivatives. *J. Am. Chem. Soc.* **76**, 2357-2362 (1954).

22 Wang, Y. & Sauer, D. R. Use of polymer-supported Pd reagents for rapid and efficient Suzuki reactions using microwave heating. *Org. Lett.* **6**, 2793-2796 (2004).

23 Mor, M. *et al.* Cyclohexylcarbamic acid 3 ‘-or 4 ‘-substituted biphenyl-3-yl esters as fatty acid amide hydrolase inhibitors: Synthesis, quantitative structure− activity relationships, and molecular modeling studies. *J. Med. Chem.* **47**, 4998-5008 (2004).

24 Zhang, L. & Wu, J. Palladium-catalyzed Hiyama cross-couplings of aryl arenesulfonates with arylsilanes. *J. Am. Chem. Soc.* **130**, 12250-12251 (2008).

25 Itoh, Y., Brossi, A., Hamel, E. & Lin, C. M. Colchicine Models: Synthesis and Binding to Tubulin of Tertamethoxybiphenyls. *Helv. Chim. Acta* **71**, 1199-1209 (1988).

26 Kataoka, N., Shelby, Q., Stambuli, J. P. & Hartwig, J. F. Air stable, sterically hindered ferrocenyl dialkylphosphines for palladium-catalyzed C− C, C− N, and C− O bond-forming cross-couplings. *J. Org. Chem.* **67**, 5553-5566 (2002).
